# A Rigid–Foldable Kirigami‐Inspired Metamorphic Mechanism With Programmable Motion Modes and Self‐Locking

**DOI:** 10.1002/advs.76798

**Published:** 2026-08-06

**Authors:** Jianlin Wang, Zhongmin Song, Ketao Zhang

**Affiliations:** ^1^ Centre for Advanced Robotics (ARQ) Queen Mary University of London London UK; ^2^ Institute of Artificial Intelligence, School of Future Technology Shanghai University Shanghai China

**Keywords:** kirigami structure, metamorphic mechanisms, multimodal motion, programmable structure, self‐locking mechanism

## Abstract

Compact metamorphic mechanisms capable of multifunctionality, especially geometric‐constraint‐induced changes in motion mode and intrinsic self‐locking without sustained actuation, are valuable for engineering deployable and adaptive robotic systems. This paper presents a rigid–foldable, kirigami‐inspired metamorphic parallel mechanism composed solely of revolute joints. Based on screw theory, we establish a unified kinematic model that systematically characterizes the four distinct configurations and their corresponding motion modes, including one uniaxial twisting motion, two coupled translations (lateral–vertical and longitudinal–vertical), and a self‐locking state consistent with the motion characteristics of the rigid–foldable kirigami. The analysis reveals that the mechanism can switch among the four motion modes by reconfiguring the geometric constraints through changes in the programming pattern of the four actuated joints, without reassembly of the kinematic structure. The proof‐of‐concept prototype and experimental tests validate motion‐mode switching and the passive structural rigidity of the tested energy‐free self‐locking configuration. This work demonstrates a way to encode functional adaptability directly into robot body design, reducing reliance on complex actuation and control systems. Such principles have broad implications for scalable, energy‐efficient systems in fields ranging from robotics and aerospace structures to biomedical devices.

## Graphical abstract

Geometric constraint reconfiguration enables a rigid–foldable kirigami‐inspired mechanism to switch between twisting, directional translation, and self‐locking states. By encoding multifunctionality directly into its architecture, the mechanism achieves multimodal motion and passive locking without reassembly, providing a compact platform for adaptive robotic and deployable systems.

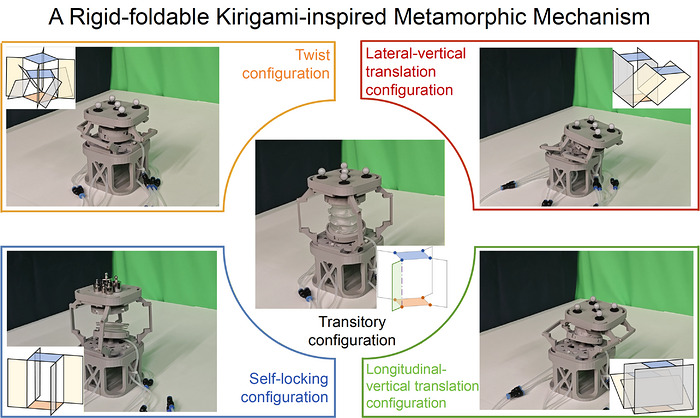

## 1 Introduction

Reconfigurable mechanical systems, such as metamorphic mechanisms capable of adapting their kinematic functions, are central to emerging applications in multifunctional robotics, deployable structures, and biomedical devices [1, 2, 3, 4, 5]. A key challenge in designing such reconfigurable systems is the integration of multiple motion modalities within a single compact mechanism, enabling transitions between distinct motion modes such as translation, rotation, and locking without reassembly of the mechanism. Within this research trend, reconfigurable mechanisms are investigated using the conventional kinematic synthesis approaches for sophisticated kinematic architectures, such as overconstrained linkages, closed‐loop hybrid linkages, modular parallel mechanisms, deployable single‐loop mechanisms, and others, to realize mobility change and motion mode switching [6, 7, 8, 9]. Recent studies in origami and kirigami engineering offer a fundamentally different paradigm, in which kinematics and mechanical performance are encoded directly in the topology and geometry of crease patterns. This geometric encoding facilitates compact mechanisms with deployability, foldability, and self‐locking, achieved without additional mechanical elements [10, 11, 12, 13, 14, 15, 16, 17].

Within this design paradigm, rotational and symmetric patterns are especially attractive as kinematic building blocks, as they enable coordinated global deformation governed by a small number of parameters while maintaining structural compactness [18, 19, 20, 21, 22]. Representative examples include twisted tower structures, which exhibit spring‐like extension/contraction coupled with controllable twist [23, 24], and Kresling‐type geometries, which demonstrate pronounced compression–torsion coupling and intrinsic multistability [18, 25]. More recent studies further revealed that chirality and geometric coupling can be enriched within a single module by combining rigid and nonrigid subpatterns [22, 26]. Twisting skeleton mechanisms exploit the same principle by converting linear input into rotary motion through fold‐inspired spatial linkages [27, 28]. Beyond coupled screw motion, modular chiral origami assemblies have demonstrated actuation‐dependent reorganization of axial displacement and rotation into distinct deformation modes at the assembly level, thereby expanding the design space for axial–torsional programmability [29].

A parallel line of research has explored origami‐ and kirigami‐inspired modules that move beyond a single helical mode to offer a repertoire of distinct deformation modes and their combinations. Representative designs include fold‐patterned building blocks that embed multiple basic modes within a single geometric module, demonstrating that motion diversity can be programmed into the pattern itself rather than introduced through hardware substitution [30, 31, 32, 33, 34]. Reconfigurability can also be achieved by modulating the effective joint states within each unit so that the same assembly can realize different shapes and motions [35, 36]. In addition to geometric design, fabrication‐aware workflows have become increasingly important for translating origami and kirigami patterns into reliable mechanisms. Additive manufacturing enables customized joints with tunable nonlinear behavior [37], while folding‐assembly, fabric‐stacking, and monolithic printing strategies provide scalable and repeatable routes to integrated three‐dimensional folding structures across platforms and length scales [38, 39, 40].

In addition to multimodal motion, many applications require a robust self‐locking state to provide load‐bearing stiffness or energy‐free holding [41, 42, 43, 44, 45]. Origami structures can meet this need through geometric multistability and constraint‐induced self‐locking. For example, perpendicular folding can stiffen a foldable structure and create a self‐locking deployed state while preserving the ability to fold flat [42]. Opposite‐folding double‐layer architectures further exploit interlocking between layers to increase bending stiffness after deployment, enabling flat‐packable structures that transform into load‐bearing shapes [43]. With thickness‐accommodation techniques, fold patterns and modular units equipped with passive elements can also be designed to exhibit programmable multistability, allowing discrete stable states to be selected by design rather than external latching [44]. Such principles have been extended to applications including origami‐based exoskeletons, where multistability enables long‐stroke transitions combined with high stiffness in stable configurations [45].

Despite these advances, there are very limited reconfigurable mechanisms that unify not only multiple motion modes but also energy‐free self‐locking states within a single kinematic structure. In addition, the principles that govern how constraint changes reconfigure mobility, as well as the methods for implementing such reconfiguration, particularly the command and regulation of transitions among motion modes, have not yet been fully investigated.

In this work, we present a programmable kirigami‐inspired metamorphic mechanism that integrates translations, helical twisting, and self‐locking within a single kinematic structure. By applying reorientation rules to the hinge–chain layout, the geometric constraints of the mechanism can be reprogrammed, thereby reconfiguring its global mobility and enabling distinct, repeatable kinematic behaviors. The main contributions of this work are as follows:

(1) A kirigami‐inspired metamorphic mechanism capable of four distinct working modes, including uniaxial twist, two translations with programmable direction, and an energy‐free self‐locking state, with no need for reassembly of the kinematic structure.
(2) A unified kinematic model that reveals the geometric constraint variations that characterize the distinct twist, translation, and self‐locking modes.
(3) A configuration‐programming strategy based on geometric constraint changes, enabling switching between working modes through selective actuation patterns of programming angles.
(4) Experimental validation of the predicted motion modes and self‐locking behavior through prototype demonstrations and quantitative characterization, confirming agreement with the theoretical analysis.

## 2 A Rigid–Foldable Kirigami

**FIGURE 1:**
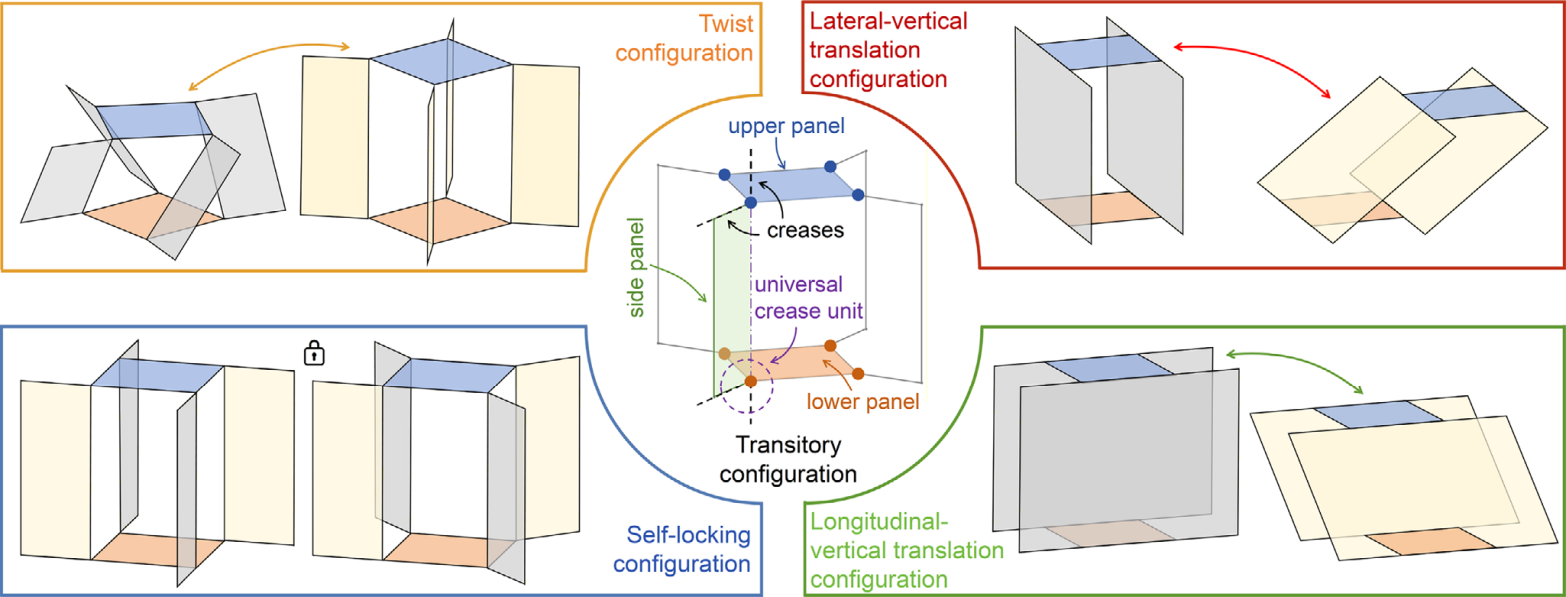
Conceptual overview of the kirigami‐inspired reconfigurable structure. The central schematic shows the transitory configuration, in which two rigid panels are connected by four side panels generated by selective lateral cuts. Reorienting the side panels reprograms the global constraint set and enables the representative twist, translation, and self‐locking configurations.

The schematic diagram illustrating a spatial rigid–foldable kirigami [27] and its configuration‐dependent motion modes is shown in Figure 1. The kirigami is obtained via a kirigami transformation of the lateral fold network, in which strategic cuts are introduced on the side surfaces to break the closed‐loop connectivity and remove redundant constraints. It consists of two identical square panels, referred to as the upper and lower panels, and four rectangular side panels. Each side panel is connected to both the lower and upper panels through a pair of orthogonal creases, referred to as a universal crease unit. Therefore, each side panel contains two universal crease units. The central schematic represents the transitory configuration of the reconfigurable structure, which serves as the common configuration for switching among the different configurations. Through programmable control of side panels, the constraint pattern of the structure can be altered, enabling the same kirigami structure to transform among different configurations. Four representative configurations can be obtained, including a twist, a longitudinal–vertical translation, a lateral–vertical translation, and a self‐locking configuration. The self‐locking configuration further includes two representative sub‐configurations: the nonorthogonal self‐locking configuration (nonsymmetric) and the orthogonal self‐locking configuration (symmetric).

**FIGURE 2:**
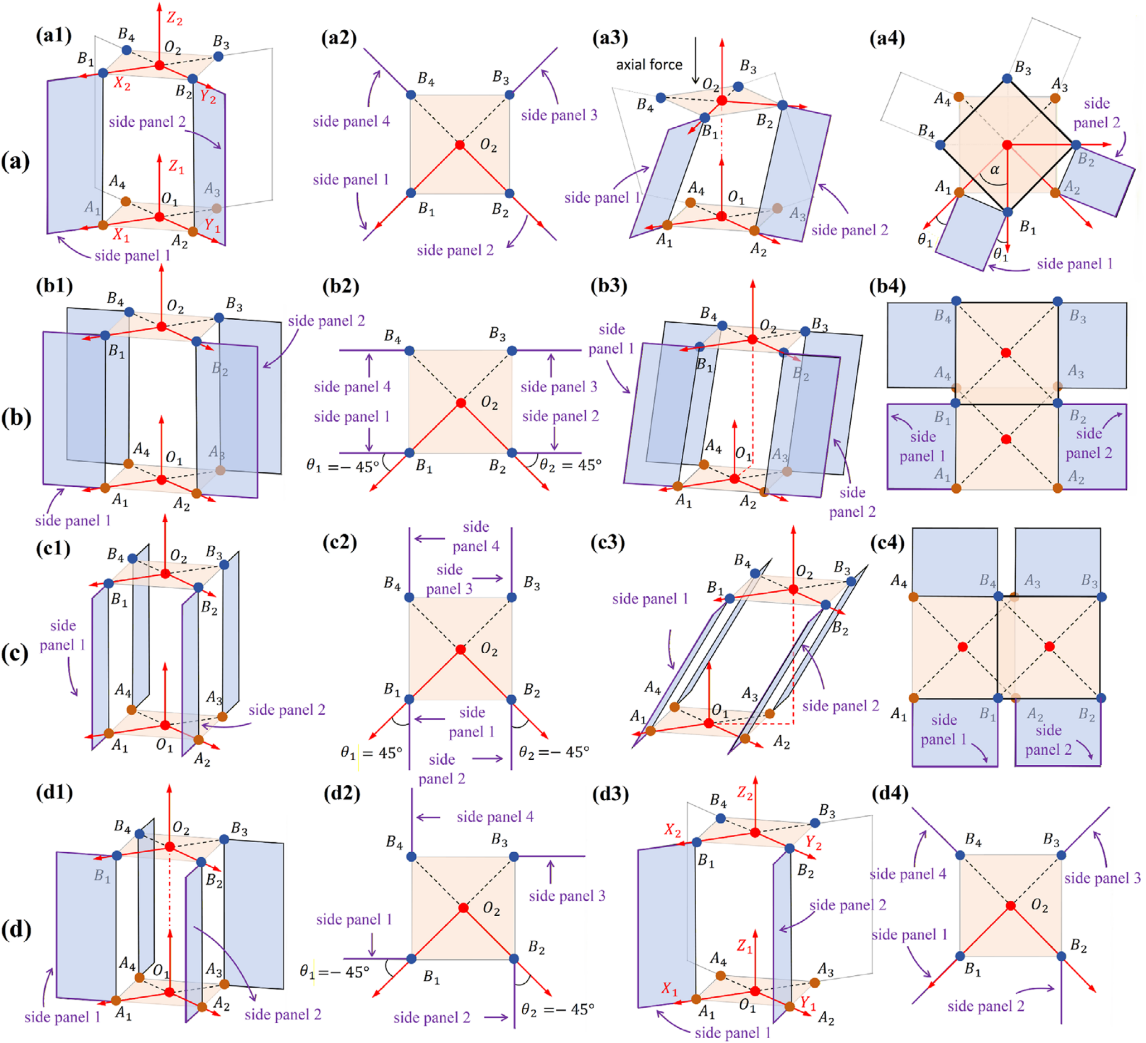
Configuration‐dependent deployment modes of the structure. Odd‐numbered subpanels show perspective views, and even‐numbered subpanels show the corresponding top views. Rows show the programmed states for (a) twist, (b) longitudinal–vertical translation, (c) lateral–vertical translation, and (d) self‐locking configurations.

To systematically describe the reconfiguration of each configuration, we introduce the reference frames and geometric parameters as shown in Figure 2. The illustration is organized row‐wise according to the four distinct configurations, and each spatial configuration is accompanied by a top view that explicitly visualizes the side panel geometric orientation pattern. The lower and upper panels are denoted by $A_{1}A_{2}A_{3}A_{4}$ and $B_{1}B_{2}B_{3}B_{4}$, respectively, where $O_{1}$ and $O_{2}$ are the geometric centers of the square panels. A Cartesian coordinate frame $O_{1}\;-\;X_{1}Y_{1}Z_{1}$ is attached to $O_{1}$ with $Z_{1}$ normal to the lower panel, and a moving frame $O_{2}\;-\;X_{2}Y_{2}Z_{2}$ is attached to $O_{2}$. The lower panel is defined as the fixed base, and the motion of the upper panel is described with respect to $O_{1}\;-\;X_{1}Y_{1}Z_{1}$. The four side panels, denoted by panels 1, 2, 3, and 4, connect to the lower and upper panels at $A_{1}$ and $B_{1}$, $A_{2}$ and $B_{2}$, $A_{3}$ and $B_{3}$, and $A_{4}$ and $B_{4}$, respectively. The orientation of each side panel can be programmed by adjusting the two pairs of universal crease units. We characterize this programming by the reorientation angles $\theta_{1},\theta_{2},\theta_{3},\theta_{4}$, defined as the angles between the reference diagonals $O_{1}A_{1}$, $O_{1}A_{2}$, $O_{1}A_{3}$, $O_{1}A_{4}$, and the corresponding side panel creases as illustrated in the top view.

**FIGURE 3:**
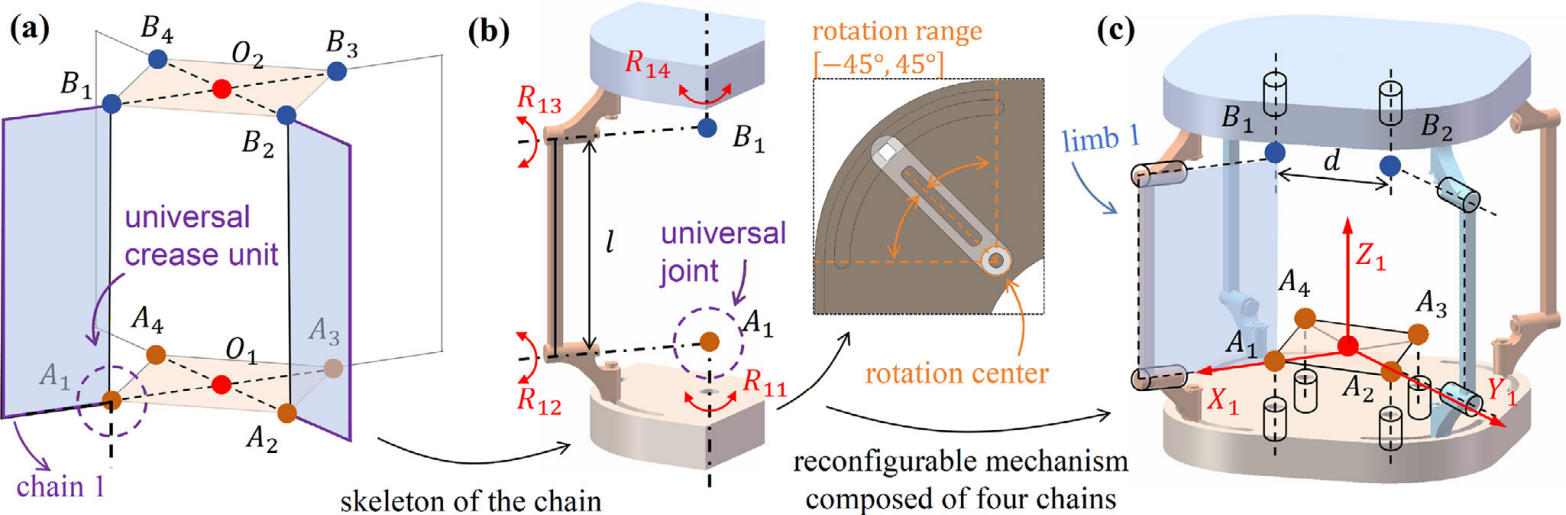
Kinematic equivalent abstraction for the reconfigurable mechanism. (a) Conceptual mechanism with two rigid panels and four limbs. (b) Skeleton of limb 1, showing the revolute joint axes $R_{11}$–$R_{14}$, the characteristic limb length l, and the admissible programming range $\theta_{1}\in \left[-45^{\circ },\;45^{\circ }\right]$. (c) Mechanism‐level skeleton with four identical limbs, platform side length d and the base frame $O_{1}\;-\;X_{1}Y_{1}Z_{1}$.

In the transitory configuration as illustrated in Figure 2a‐1,2, the planes of adjacent side panels are mutually orthogonal and define a line‐symmetric reference state. This reference state provides the geometric basis for subsequent deployment and mode switching. All programming angles $\theta_{1}=\theta_{2}=\theta_{3}=\theta_{4}=0^{\circ }$, as shown in Figure 2a‐1. Under an external force applied to the upper panel, the structure evolves from the transitory configuration into a twist‐dominated configuration, as shown in Figure 2a‐3,4. In this configuration, all four side panels are line‐symmetric with respect to the axis formed by the geometric centers ($O_{1}$ and $O_{2}$) of the upper and lower panels, and the upper panel implements a helical motion about the central axis. The relative angular displacement between the two panels about $Z_{1}$ is denoted by α. The corresponding programming angle is denoted by $\theta_{1}$ (and similarly $\theta_{2}$), where $\alpha =2\theta_{1}$ in this symmetric deployment.

**FIGURE 4:**
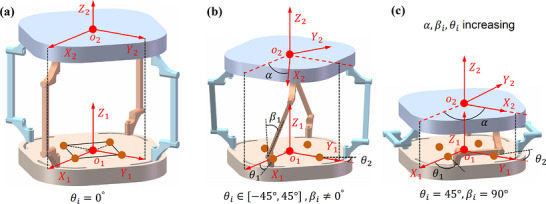
Kinematic evolution of the twist configuration. (a) Transitory configuration, which is also the home position of the twist configuration. (b) Finite twist under axial input with $\theta_{1}=\theta_{2}=\theta_{3}=\theta_{4}=\theta$, and $\alpha =2\theta \neq 0^{\circ }$. (c) Limiting twisted state imposed by the mechanical programming range $\theta \in [-45^{\circ },45^{\circ }]$, corresponding to $\alpha \in [-90^{\circ },90^{\circ }]$.

Following the same transitory logic, the translational motion modes are also obtained from the transitory configuration. Unlike the twist mode, the side panels are rearranged into two pairs, with each pair consisting of two adjacent coplanar panels. The resulting translation direction is determined by the specific pair of side panels that satisfies the coplanar condition, leading to either longitudinal (forward–backward)–vertical (Figure 2b‐1–4) or lateral (left–right)–vertical (Figure 2c‐1–4) translation. In the configuration shown in Figure 2b‐2, the programming angles $\theta_{1}=-45^{\circ }$ and $\theta_{2}=45^{\circ }$, meaning that side panels 1 and 2 are coplanar and the same for side panels 3 and 4. This leads to the translation along the longitudinal direction. In the configuration shown in Figure 2c‐2, the alternative programming angles $\theta_{1}=45^{\circ }$ and $\theta_{4}=-45^{\circ }$, and side panels 1 and 4 are coplanar. This leads to the translation along one lateral direction. Figure 2b‐1 shows the home position of the longitudinal–vertical translation configuration. This home position is a geometric transitory configuration from which the mechanism can move either forward or backward. Figure 2c‐1 shows the home position of the lateral–vertical translation configuration, from which the mechanism can move either leftward or rightward.

When the programmed orientations of the side panels satisfy neither the twist condition nor the coplanar condition for the translation modes, the structure can enter a self‐locking configuration through two different principles as shown in Figure 2d‐1–4. In the nonorthogonal self‐locking configuration, the locking is governed by geometric constraints. In the orthogonal self‐locking configuration, the locking is governed by the combined effect of geometric constraints and mechanical constraints. It is realized when all programmed side panels reach their designed boundary limits. At these limits, the structural stops engage and prevent further admissible motion. In Figure 2d‐1–2, when the four programming angles satisfy $\theta_{1}=\theta_{2}=\theta_{3}=\theta_{4}=\pm 45^{\circ }$, the adjacent side panels become pairwise orthogonal, and the kirigami turns to a structure that sustains axial loading along the axis passing through geometric centers $O_{1}$ and $O_{2}$. In addition to the orthogonal self‐locking arrangement, Figure 2d‐3–4 illustrates one representative nonorthogonal self‐locking configuration, which is generated by reorienting one side panel relative to the transitory configuration. More generally, non‐orthogonal self‐locking can be obtained when the programmed orientations of the side panels form an asymmetric pattern that satisfies neither the twist configuration nor the coplanar condition for the translation configuration. This asymmetric reorientation pattern introduces additional geometric constraints that suppress the global motion of the kirigami, yielding a structure that can also sustain axial force along the axis.

For each programmed motion configuration, we define a home position as the configuration‐specific reference pose from which the associated finite motion is initiated. The home positions of the twist, longitudinal–vertical translation, and lateral–vertical translation configurations are shown in Figure 2a‐1, Figure 2b‐1, and Figure 2c‐1, respectively. Each of these home positions is a geometric transitory configuration at which two opposite motion branches meet. The twist home position in Figure 2a‐1 coincides with the transitory configuration, whereas the translation home positions in Figure 2b‐1 and 2c‐1 are configurations distinct from the transitory configuration. The transitory configuration is the common intermediate for switching between different configurations.

## 3 Kinematic Modeling of the Kirigami‐Inspired Metamorphic Mechanism

### 3.1 Kinematic Equivalent Abstraction

The kirigami introduced in Section 2 illustrates intrinsic capabilities in changing configuration and motion mode by programming geometric conditions between side panels. In this section, we abstract the kirigami structure into an equivalent rigid‐link mechanism and apply the fundamentals of kinematics modeling to reveal the underlying principle of the configuration changes of the kirigami. The symbols and notation used in the following analysis are summarized in Table 1.

**TABLE 1:** Nomenclature for the screw‐theoretic formulation.

Symbol	Description	Unit
$O_{1}\;-\;X_{1}Y_{1}Z_{1}$	Base coordinate frame attached to the fixed platform	—
$O_{2}\;-\;X_{2}Y_{2}Z_{2}$	Moving coordinate frame attached to the top platform	—
$n_{i}$	Pure‐couple constraint direction / normal vector of the corresponding limb plane	—
$A_{i}$	Position vectors of $A_{i}$ expressed in $O_{1}\;-\;X_{1}Y_{1}Z_{1}$	mm
$B_{i}$	Position vectors of $B_{i}$ expressed in $O_{1}\;-\;X_{1}Y_{1}Z_{1}$	mm
$p={\left[p_{x},p_{y},p_{z}\right]}^{T}$	Translation of $O_{2}$ relative to $O_{1}$, expressed in $O_{1}\;-\;X_{1}Y_{1}Z_{1}$	mm
d	Side length of the square platform layout, with $d=∥A_{1}A_{2}∥=∥A_{2}A_{3}∥$	mm
r	Radial distance from $O_{1}$ to each lower attachment point, with $r=d/\sqrt{2}$	mm
l	Distance between the two limb attachment points, with $l=∥A_{i}B_{i}∥$	mm
$\varphi_{i}$	Fixed reference angle of limb i, measured from the $X_{1}$‐axis	deg
$\theta_{i}$	Programming angle of the horizontal joint axes	deg
$\Theta =[\theta_{1},\theta_{2},\theta_{3},\theta_{4}]$	Vector of the four programming angles	deg
$\psi_{i}$	Effective joint‐axis angle of limb i, defined as $\psi_{i}=\varphi_{i}+\theta_{i}$	deg
$\beta_{i}$	Limb deflection angle of limb i	deg
α	Relative yaw rotation of the top platform with respect to the base about $Z_{1}$	deg
$R_{ij}$	Revolute joint axis j in limb i	—
$\omega_{\mathrm{ij}}$	Unit direction vector of revolute joint axis $R_{ij}$	—
$v_{\mathrm{ij}}$	Linear component of the unit twist screw	—
$S_{ij}$	Twist screw of joint axis $R_{ij}$ in Plücker coordinates	—
$S_{i}^{r}$	Constraint wrench of limb i	—
$S^{r}$	Full global constraint matrix assembled from all limbs	—
$S^{m}$	Platform motion screw system	—
$S_{tw}^{r}$	Constraint screw system in the twist configuration	—
$S_{tr}^{r}$	Constraint screw system in the translation configuration	—
$C_{o}$	Transitory configuration set	—
$C_{tw}$	Twist configuration set	—
$C_{tr}$	Translation configuration set	—
$C_{lk}$	Self‐locking configuration set	—

*Note*: The limb index is i∈{1,2,3,4} and the joint index is j∈{1,2,3,4}.

Taking panels as rigid links and creases as revolute joints, the equivalent kinematic abstraction of the kirigami is summarized in Figure 3. The universal crease unit, shown in Figure 3a, is abstracted to a universal joint (Hooke joint) with two orthogonal revolute joints as illustrated in Figure 3b. The lower and upper panels are modeled as rigid platforms, denoted by $A_{1}A_{2}A_{3}A_{4}$ and $B_{1}B_{2}B_{3}B_{4}$, respectively. Both platforms are characterized by the side length d (e.g., $d=∥A_{1}A_{2}∥=∥A_{2}A_{3}∥$) of the squares defined by the attachment points of the universal joints. The two universal joints together with the rigid link between them form a serial kinematic limb with four‐revolute (4R) joints with axes $R_{11}$ and $R_{12}$ intersecting at point $A_{1}$ and $R_{13}$ and $R_{14}$ intersecting at $B_{1}$. Each limb is characterized by one key design parameter, $l=∥A_{1}B_{1}∥=∥A_{2}B_{2}∥$, the distance between the two universal joints. As such, the kinematic equivalent of the kirigami is a parallel mechanism (Figure 3c) consisting of four identical kinematic chains, each connecting the upper and lower platforms via universal joints. For limb i, the four revolute joint axes are denoted by $R_{ij}$, where i∈{1,2,3,4} and j∈{1,2,3,4}. The second subscript j follows the order along limbs from the lower platform to the upper platform: $R_{i1}$ is the revolute axis adjacent to the lower platform, followed by $R_{i2}$ and $R_{i3}$, while $R_{i4}$ is the revolute axis adjacent to the upper platform. Accordingly, $R_{i1}$ and $R_{i2}$ intersect at $A_{i}$, whereas $R_{i3}$ and $R_{i4}$ intersect at $B_{i}$. The programmed reorientation of limb i is bounded by mechanical stops at the platform‐attached joints with axes $R_{i1}$ and $R_{i4}$, defining the admissible range as $\theta_{i}\in \left[-45^{\circ },45^{\circ }\right]$. With design parameters (d,l) and the programming angles $\left\{\theta_{1},\theta_{2},\theta_{3},\theta_{4}\right\}$ specified, the spatial location of all revolute axes can be fully formulated in terms of screw theory in the base frame $O_{1}\;-\;X_{1}Y_{1}Z_{1}$, attached to the lower platform with the same setting as defined in Section 2.

### 3.2 Screw Twist and Constraint Analysis

This section formulates the constraints of the metamorphic parallel mechanism based on screw theory. A twist is expressed in Plücker coordinates as

(1) $$S=\left[\begin{array}{c} v\\ \omega \end{array}\right]\in R^{6}$$

where v and ω denote the linear and angular velocity components. A wrench (constraint screw) is expressed as

(2) $$S^{r}=\left[\begin{array}{c} f\\ \tau \end{array}\right]\in R^{6}$$

where f is the force and τ is the moment about the origin of $O_{1}\;-\;X_{1}Y_{1}Z_{1}$. The wrench $S^{r}$ and twist S are reciprocal to one another if their reciprocity product satisfies

(3) $$S^{r}⊙S=f^{T}v+\tau^{T}\omega =0.$$

Let $A_{i}$ and $B_{i}$ denote the common points of the universal joints in limb i connected to the lower and upper platforms, respectively. In the base frame $O_{1}-X_{1}Y_{1}Z_{1}$, the position vectors of the common points $A_{i}$ are denoted by $A_{i}$ and given by

(4) $$\begin{array}{cc} A_{1} & ={\left(r,0,0\right)}^{T},\;A_{2}={\left(0,r,0\right)}^{T},\\ A_{3} & ={\left(-r,0,0\right)}^{T},\;A_{4}={\left(0,-r,0\right)}^{T}. \end{array}$$

Define $e_{x}={\left[1,0,0\right]}^{T}$, $e_{y}={\left[0,1,0\right]}^{T}$, and $e_{z}={\left[0,0,1\right]}^{T}$. Let $\varphi_{i}$ denote the angles between $O_{1}A_{i}$ (i = 1,2,3 and 4) and $X_{1}$‐axis, this gives $\varphi_{1}=0^{\circ },\varphi_{2}=90^{\circ },\varphi_{3}=180^{\circ }$, and $\varphi_{4}=270^{\circ }$. The programming angle $\theta_{i}$ reorients the plane, defined by the two parallel axes of joints $R_{i2}$ and $R_{i3}$, relative to this reference direction shown in Figure 3c. Hence, the orientation of the programmable revolute joint ($R_{i1}$) is determined by both the fixed reference angle $\varphi_{i}$ and the programming angle $\theta_{i}$, that is

(5) $$\psi_{i}=\varphi_{i}+\theta_{i}.$$

The position vectors of $B_{i}$, the common points of universal joints connecting to the upper platform in limb i, are given by

(6) $$B_{i}=R_{z}\left(\alpha \right)\left[\begin{array}{c} x_{i}\\ y_{i}\\ 0 \end{array}\right]+p=\left[\begin{array}{c} x_{i}\cos\alpha -y_{i}\sin\alpha +p_{x}\\ x_{i}\sin\alpha +y_{i}\cos\alpha +p_{y}\\ p_{z} \end{array}\right].$$

Here, $R_{z}\left(\alpha \right)$ is the rotation matrix about $Z_{1}$, and the relative pose of the upper platform is described by the yaw angle α and the translation vector $p={\left[p_{x},p_{y},p_{z}\right]}^{T}$. The vector $\omega_{ij}$ denotes the unit vector of joint axis $R_{ij}$. Expressed in $O_{1}\;-\;X_{1}Y_{1}Z_{1}$, they are given by

(7) $$\omega_{i1}=\omega_{i4}=e_{z},\;\omega_{i2}=\omega_{i3}=R_{z}\left(\psi_{i}\right)e_{x}=\left[\begin{array}{c} \cos\psi_{i}\\ \sin\psi_{i}\\ 0 \end{array}\right].$$

The unit normal to $\omega_{i2}$ is then expressed as

(8) $$R_{z}\left(\psi_{i}\right)e_{y}=\left[\begin{array}{c} -\sin\psi_{i}\\ \cos\psi_{i}\\ 0 \end{array}\right].$$

With the unit vectors $\omega_{ij}$ and position vectors $A_{i}$ and $B_{i}$, the twist screws of the four joints in limb i are explicitly given as

(9) $$\begin{array}{c} S_{i1}=\left[\begin{array}{c} y_{i}\\ -x_{i}\\ 0\\ 0\\ 0\\ 1 \end{array}\right],\;S_{i2}=\left[\begin{array}{c} 0\\ 0\\ x_{i}\sin\psi_{i}-y_{i}\cos\psi_{i}\\ \cos\psi_{i}\\ \sin\psi_{i}\\ 0 \end{array}\right],\\ S_{i3}=\left[\begin{array}{c} -p_{z}\sin\psi_{i}\\ p_{z}\cos\psi_{i}\\ \left(x_{i}\cos\alpha -y_{i}\sin\alpha +p_{x}\right)\sin\psi_{i}\\ \;-\left(x_{i}\sin\alpha +y_{i}\cos\alpha +p_{y}\right)\cos\psi_{i}\\ \cos\psi_{i}\\ \sin\psi_{i}\\ 0 \end{array}\right],\\ S_{i4}=\left[\begin{array}{c} x_{i}\sin\alpha +y_{i}\cos\alpha +p_{y}\\ -(x_{i}\cos\alpha -y_{i}\sin\alpha +p_{x})\\ 0\\ 0\\ 0\\ 1 \end{array}\right] \end{array}$$

and the limb motion screw system is

(10) $$S_{i}=\left\{S_{i1},S_{i2},S_{i3},S_{i4}\right\}\in R^{6\times 4}.$$

The constraint system transmitted by limb i is a set of wrenches, $S^{r}$, satisfying ${S^{r}}^{T}S_{i}=0$. For each limb, there are two independent wrenches, which are expressed as

(11) $$S_{i1}^{r}=\left[\begin{array}{c} B_{i}-A_{i}\\ A_{i}\times \left(B_{i}-A_{i}\right) \end{array}\right],\;S_{i2}^{r}=\left[\begin{array}{c} 0\\ R_{z}\left(\psi_{i}\right)e_{y} \end{array}\right].$$

Substituting the position vectors into Equation (11), the constraint wrenches for the four limbs are explicitly given as

(12) $$\begin{array}{ccc} & & S_{11}^{r}=\left[\begin{array}{c} p_{x}+r\left(\cos\alpha -1\right)\\ p_{y}+r\sin\alpha \\ p_{z}\\ 0\\ -rp_{z}\\ rp_{y}+r^{2}\sin\alpha \end{array}\right],\;S_{12}^{r}=\left[\begin{array}{c} 0\\ 0\\ 0\\ -\sin\theta_{1}\\ \cos\theta_{1}\\ 0 \end{array}\right],\\ & & S_{21}^{r}=\left[\begin{array}{c} p_{x}-r\sin\alpha \\ p_{y}+r\left(\cos\alpha -1\right)\\ p_{z}\\ rp_{z}\\ 0\\ -r(p_{x}-r\sin\alpha ) \end{array}\right],\;S_{22}^{r}=\left[\begin{array}{c} 0\\ 0\\ 0\\ -\cos\theta_{2}\\ -\sin\theta_{2}\\ 0 \end{array}\right],\\ & & S_{31}^{r}=\left[\begin{array}{c} p_{x}+r\left(1-\cos\alpha \right)\\ p_{y}-r\sin\alpha \\ p_{z}\\ 0\\ rp_{z}\\ -r(p_{y}-r\sin\alpha ) \end{array}\right],\;S_{32}^{r}=\left[\begin{array}{c} 0\\ 0\\ 0\\ \sin\theta_{3}\\ -\cos\theta_{3}\\ 0 \end{array}\right],\\ & & S_{41}^{r}=\left[\begin{array}{c} p_{x}+r\sin\alpha \\ p_{y}+r\left(1-\cos\alpha \right)\\ p_{z}\\ -rp_{z}\\ 0\\ r(p_{x}+r\sin\alpha ) \end{array}\right],\;S_{42}^{r}=\left[\begin{array}{c} 0\\ 0\\ 0\\ \cos\theta_{4}\\ \sin\theta_{4}\\ 0 \end{array}\right]. \end{array}$$

Stacking the four limbs yields the full global constraint system

(13) $$S^{r}=\left\{S_{11}^{r},S_{12}^{r},S_{21}^{r},S_{22}^{r},S_{31}^{r},S_{32}^{r},S_{41}^{r},S_{42}^{r}\right\}\in R^{6\times 8}.$$

### 3.3 Variation in Constraint System and Motion Mode

#### 3.3.1 Transitory Configuration

Based on the unified screw theory formulation in Section 3.2, different combinations of the programming angles $\theta_{i}$ change the global constraint screw system $S^{r}$, and consequently the admissible platform motion screw system $S^{m}$. To describe the programmed reconfigurations in a compact form, we define a vector composed of all programming parameters as

(14) $$\Theta =\left[\begin{array}{cccc} \theta_{1} & \theta_{2} & \theta_{3} & \theta_{4} \end{array}\right].$$

When the mechanism is in the configuration illustrated in Figure 3c, the programming parameters $\theta_{i}=0^{\circ }$ and the platform yaw angle $\alpha =0^{\circ }$, explicitly expressed as

(15) $$C_{o}=\left\{\left(\Theta ,\alpha \right)\;|\;\theta_{1}=\theta_{2}=\theta_{3}=\theta_{4}=0^{\circ },\;\alpha =0^{\circ }\right\}.$$

This pose is termed the transitory configuration of the metamorphic parallel mechanism, corresponding to the transitory configuration of the kirigami in Figure 1. This configuration is the home position of the twist configuration. It also serves as the common intermediate for switching between any two different operation modes. In the following, we analyze how variations in Θ modify the constraint system and lead to the representative twist (or helical motion), direction‐programmed translation, and self‐locking configurations.

#### 3.3.2 Twist Configuration

When an external load is applied to the upper platform, the parallel mechanism is able to reconfigure from the transitory configuration to the configurations in Figure 4 with helical motion about the central axis without actively changing the programming angles of joints $R_{i1}$. Along the finite twist movement, the four programming angles remain identical as $\theta_{1}=\theta_{2}=\theta_{3}=\theta_{4}=\theta$ and evolve passively under the axial input, while the moving platform yaw angle satisfies α=2θ. When $\theta =0^{\circ }$ and the rotation angle of the upper platform $\alpha =0^{\circ }$, the mechanism returns to the transitory configuration in Figure 4a. The twist states are

(16) $$C_{\mathrm{tw}}=\left\{\left(\Theta ,\alpha \right)\;|\;\theta_{1}=\theta_{2}=\theta_{3}=\theta_{4}=\theta ,\;0<\left|\theta \right|\leq 45^{\circ },\;\alpha =2\theta \right\}.$$

Since the programming angle is mechanically bounded within $\theta \in [-45^{\circ },\;45^{\circ }]$ to avoid collision between limbs, the corresponding platform yaw angle is bounded within $\alpha \in [-90^{\circ },\;90^{\circ }]$. Substituting the parameters in Equation (16) into the limb constraint wrenches in Equation (13), the global constraint screw system for the twist configuration is obtained as

(17)

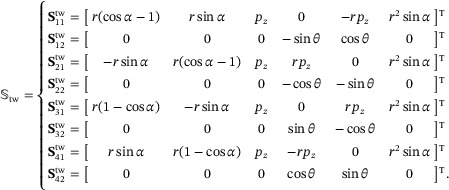




According to the screw‐theory based reciprocal product defined in Equation (3), the motion screw system, $S_{\mathrm{tw}}^{m}$ of the mechanism is calculated as

(18) $$S_{\mathrm{tw}}^{m}=S^{m}={\left[\begin{array}{cccccc} 0 & 0 & -\frac{r^{2}\sin\alpha }{p_{z}} & 0 & 0 & 1 \end{array}\right]}^{T},\;p_{z}\neq 0.$$

Equation (18) is a screw system consisting of one screw that represents a coupled axial translation and rotation about the $Z_{1}$‐axis. An axial input normal to the platform plane is converted into a helical twist motion of the moving platform. In addition, the third component of $S_{\mathrm{tw}}^{m}$ gives the pitch of the instantaneous screw motion

(19) $$p_{\mathrm{tw}}=\frac{v_{z}}{\omega_{z}}=-\frac{r^{2}\sin\alpha }{p_{z}}.$$

**FIGURE 5:**
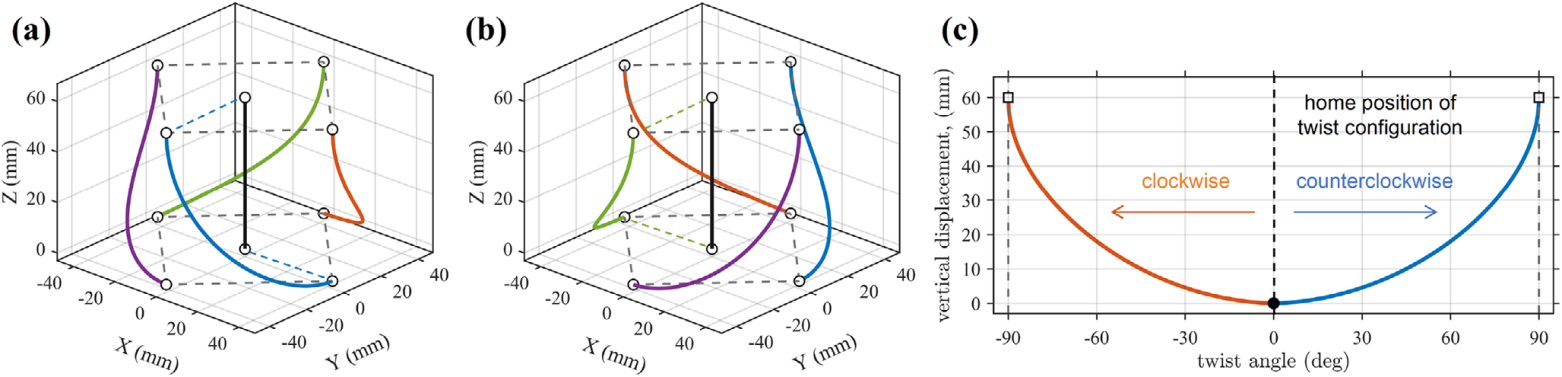
Finite‐motion trajectories of the twist configuration. (a) Counterclockwise and (b) clockwise helical motions of the upper platform. (c) Axial displacement as a function of the twist angle. The dashed line marks the home position of the twist configuration.

To further clarify the finite motion of the twist configuration, the trajectories of four peripheral points and the center of the upper platform are presented. At the home position of the twist configuration, shown in Figure 4a, the twist angle and axial displacement are both zero. The mechanism can implement clockwise or counterclockwise finite motion by passing this home position. As illustrated in Figure 5a,b, the upper‐platform center undergoes displacement only along the *Z* axis, whereas the four peripheral points follow four congruent helical trajectories. Figure 5c shows the corresponding relationship between axial displacement and twist angle. The two branches are continuous on either side of the home position. The endpoints at $\alpha =\pm 90^{\circ }$ are the ideal kinematic motion boundaries of the twist configuration.

#### 3.3.3 Translation Configuration

**FIGURE 6:**
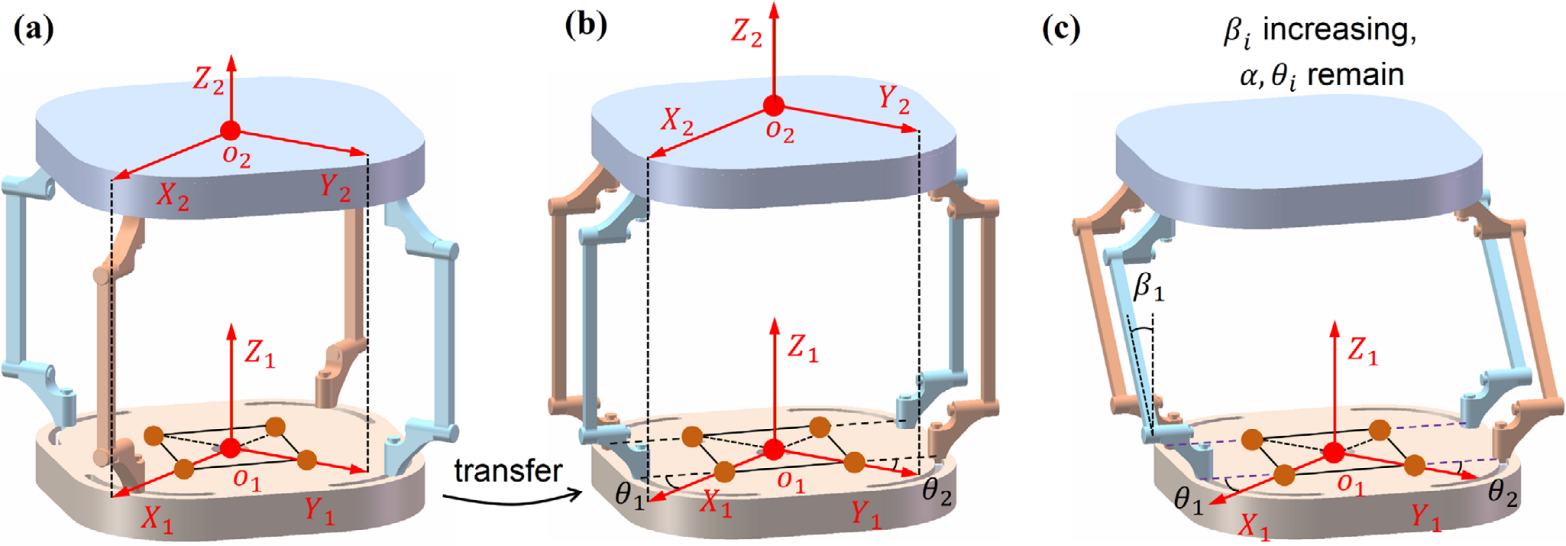
Schematic diagram of bidirectional translation configuration. (a) Transitory configuration. (b) Home position of the programmed translation configuration, with $\theta_{1}=\theta_{3}=-\theta_{2}=-\theta_{4}=-45^{\circ }$ and $\beta_{1}=\beta_{2}=\beta_{3}=\beta_{4}=0^{\circ }$. (c) Translational motion with increasing $\beta_{i}$ in the ideal kinematic range $\beta_{i}\in \left[0^{\circ },90^{\circ }\right]$, while α and $\theta_{i}$ remain fixed.

Taking two adjacent limbs as a pair, the four limbs of the parallel mechanism are then divided into two pairs. Adjusting the programming angles of joints $R_{i1}$ to enable the joints $R_{i2}$ and $R_{i3}$ within each pair to be coplanar, it allows the parallel mechanism to reconfigure from the transitory configuration in Figure 6a to a translational configuration in Figure 6b. In this configuration, the programming angles satisfy $\theta_{1}=-\theta_{2}=\theta_{3}=-\theta_{4}=\pm 45^{\circ }$, expressed in the vector form as

(20) $$C_{\mathrm{tr}}=\left\{\left(\Theta ,\alpha \right)\;|\;\theta_{1}=-\theta_{2}=\theta_{3}=-\theta_{4}=\theta_{s},\;\theta_{s}=\pm 45^{\circ },\;\alpha =0^{\circ }\right\}.$$

Substituting the geometric parameters in Equation (20) into the limb constraint wrenches expressed in Equation (13), the constraint screw system of the mechanism in this translational configuration is obtained as

(21)

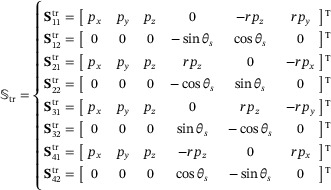




Based on the reciprocal product formulation in Equation (3) in terms of screw theory, the motion screw system of the mechanism in this translational configuration is derived as

(22) $$S_{tr}^{m}=S^{m}={\left[\begin{array}{cccccc} v_{x} & v_{y} & -\frac{p_{x}v_{x}+p_{y}v_{y}}{p_{z}} & 0 & 0 & 0 \end{array}\right]}^{T},\;p_{z}\neq 0$$

which represents a 1‐DoF (Degree of Freedom) translational motion in a single plane perpendicular to the base ($X_{1}O_{1}Y_{1}$ plane). Given that each limb has two distinct adjacent limbs, the mechanism can assume two distinct translational configurations. When joints of limbs 1 and 4 form a plane, and those of limbs 2 and 3 form another plane, the mechanism reconfigures into the lateral–vertical translation mode with $p_{x}=0,\;v_{x}=0$ and the translation described by

(23) $$S_{tr}^{m}={\left[\begin{array}{c} 0,1,-\frac{p_{y}}{p_{z}},0,0,0 \end{array}\right]}^{T},\;v_{x}=0,p_{z}\neq 0.$$

In contrast, when joints of limbs 1 and 2 form a plane, while those of limbs 3 and 4 form another plane, the mechanism reconfigures to the longitudinal‐vertical translation mode with $p_{y}=0,v_{y}=0$ and the translational motion described by

(24) $$S_{tr}^{m}={\left[\begin{array}{c} 1,0,-\frac{p_{x}}{p_{z}},0,0,0 \end{array}\right]}^{T},\;v_{y}=0,p_{z}\neq 0.$$

In these translational configurations, the programming angles are constant: $\theta_{i}=\pm 45^{\circ }$, and $\alpha =0^{\circ }$. All other passive joints align four parallel axes, enabling the translation, which can be described by the angular displacement $\beta_{i}$ as shown in Figure 6c.

**FIGURE 7:**
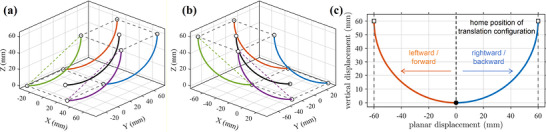
Finite‐motion trajectories of the bidirectional translation configurations. (a) Rightward motion in the lateral–vertical configuration or backward motion in the longitudinal–vertical configuration. (b) Leftward motion in the lateral‐vertical configuration or forward motion in the longitudinal–vertical configuration. (c) Planar displacement in the vertical plane. The dashed line marks the home position of the corresponding translation configuration.

To further clarify the finite motion of the translation configurations, the trajectories of four peripheral points and the center of the upper platform are presented. At the home positions of the longitudinal–vertical and lateral–vertical translation configurations, shown in Figure 2b‐1,c‐1, respectively, both the planar and vertical displacements are zero. The mechanism can translate in either of two opposite directions by passing the home position. As illustrated in Figure 7a,b, the upper platform undergoes coupled planar and vertical translation along the programmed translation axis, while the four peripheral points and the upper‐platform center follow congruent trajectories. Figure 7c shows the corresponding relationship between vertical displacement along z‐axis and planar displacement in the XY plane. In the lateral–vertical configuration, negative and positive planar displacements correspond to leftward and rightward motions, respectively. In the longitudinal–vertical configuration, they correspond to forward and backward motions, respectively. The endpoints at ±60mm are the ideal kinematic motion boundaries of the translation configurations.

#### 3.3.4 Self‐Locking Configuration

The parallel mechanism reconfigures to a self‐locking configuration whenever the programming angles of joints $R_{i1}$ satisfy

(25) $$\left(\Theta ,\alpha \right)\notin C_{o}\cup C_{\mathrm{tw}}\cup C_{\mathrm{tr}}.$$

**FIGURE 8:**
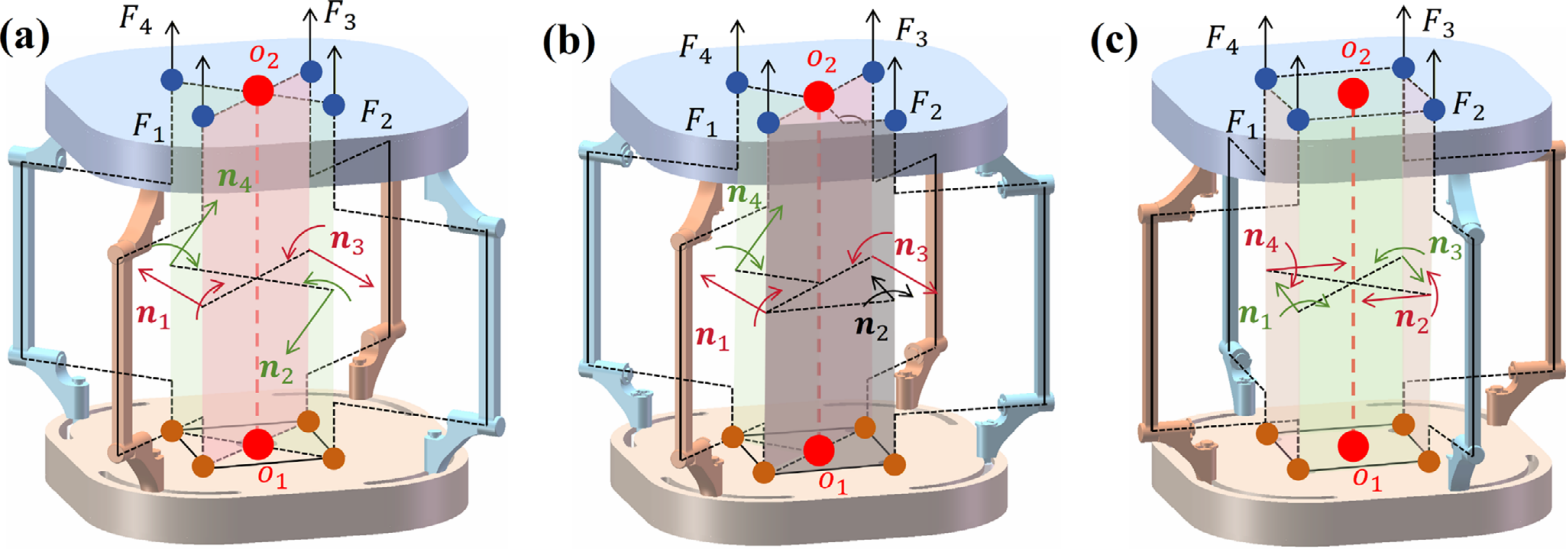
Force‐couple interpretation of the self‐locking mechanism. (a) Transitory configuration. (b) Representative nonorthogonal self‐locking configuration. (c) Orthogonal self‐locking configuration. Here $F_{1},F_{2},F_{3},F_{4}$ denote the nominal support‐reaction force lines applied to the upper platform at $B_{1},B_{2},B_{3},B_{4}$, respectively. The vectors $n_{1},n_{2},n_{3},n_{4}$ denote the pure‐couple constraint directions.

The relative relations among $\theta_{1},\theta_{2},\theta_{3},\theta_{4}$ for each limb i determine whether the resulting constraint system remains compatible with a mobile mode or evolves into a locking mode. In addition, the motion of the upper platform is constrained, and $\alpha =0^{\circ }$ when the mechanism is in a self‐locking configuration. Representative self‐locking patterns include a non‐orthogonal self‐locking configuration governed by geometric constraints and an orthogonal self‐locking configuration governed by both geometric and mechanical constraints, as shown in Figure 8b,c, respectively, defined by

(26) $$\Theta_{lk}^{\left(b\right)}=\left[\begin{array}{cccc} 0^{\circ } & -45^{\circ } & 0^{\circ } & 0^{\circ } \end{array}\right],\;\Theta_{lk}^{\left(c\right)}=\left[\begin{array}{cccc} 45^{\circ } & 45^{\circ } & 45^{\circ } & 45^{\circ } \end{array}\right].$$

Note that when the mechanism is in the orthogonal self‐locking configuration, it is not an instantaneous twist configuration and the platform yaw angle is fixed at $\alpha =0^{\circ }$, even though $\theta_{1}=\theta_{2}=\theta_{3}=\theta_{4}$. This is because the twist configuration requires α=2θ, which gives $\alpha =90^{\circ }$ when $\theta =45^{\circ }$. Therefore, these two states are distinguished. To explicitly interpret the underlying principles enabling self‐locking, Figure 8 illustrates the directions of the constraints applied by the four limbs to the upper platform. Each limb exerts an upward constraint force $F_{i}$ along $A_{i}B_{i}$. In addition to the constraint force, each limb also exerts a constraint couple on the upper platform, which is denoted by $S_{i2}^{r}$, i∈{1,2,3,4}, as described by Equation (12). For the four limbs, these constraint couples are

(27) $$\begin{array}{cc} & S_{12}^{r}\left(\theta_{1}\right)={\left[\begin{array}{cccccc} 0 & 0 & 0 & -\sin\theta_{1} & \cos\theta_{1} & 0 \end{array}\right]}^{T},\\ & S_{22}^{r}\left(\theta_{2}\right)={\left[\begin{array}{cccccc} 0 & 0 & 0 & -\cos\theta_{2} & -\sin\theta_{2} & 0 \end{array}\right]}^{T},\\ & S_{32}^{r}\left(\theta_{3}\right)={\left[\begin{array}{cccccc} 0 & 0 & 0 & \sin\theta_{3} & -\cos\theta_{3} & 0 \end{array}\right]}^{T},\\ & S_{42}^{r}\left(\theta_{4}\right)={\left[\begin{array}{cccccc} 0 & 0 & 0 & \cos\theta_{4} & \sin\theta_{4} & 0 \end{array}\right]}^{T}. \end{array}$$

The last three elements of these wrenches represent the normal vectors of the corresponding limb planes. Denoting these plane normals by $n_{i}$ for i∈{1,2,3,4}, the associated constraint couples are aligned with $n_{i}$, which are

(28) $$\begin{array}{cccc} n_{1}\left(\theta_{1}\right) & ={\left[\begin{array}{ccc} -\sin\theta_{1} & \cos\theta_{1} & 0 \end{array}\right]}^{T}, & n_{2}\left(\theta_{2}\right) & ={\left[\begin{array}{ccc} -\cos\theta_{2} & -\sin\theta_{2} & 0 \end{array}\right]}^{T},\\ n_{3}\left(\theta_{3}\right) & ={\left[\begin{array}{ccc} \sin\theta_{3} & -\cos\theta_{3} & 0 \end{array}\right]}^{T}, & n_{4}\left(\theta_{4}\right) & ={\left[\begin{array}{ccc} \cos\theta_{4} & \sin\theta_{4} & 0 \end{array}\right]}^{T}. \end{array}$$

In the transitory configuration shown in Figure 8a, limbs 1 and 3 belong to one coplanar pair, while limbs 2 and 4 belong to another, and the corresponding constraint couples satisfy

(29) $$n_{1}\left(0^{\circ }\right)=-n_{3}\left(0^{\circ }\right),\;n_{2}\left(0^{\circ }\right)=-n_{4}\left(0^{\circ }\right).$$

The constraint subsystem degenerates to two independent constraints. In this mode, the upper platform is subjected to two pairs of couples from the diagonal limbs, restricting its movement in all directions parallel to the base. When the orientation of one limb is reconfigured, the geometric conditions of the configuration change. As a result, the mechanism will enter a self‐locking configuration. One example is given by changing one programming angle $\theta_{2}$ from $0^{\circ }$ to $-45^{\circ }$, the mechanism will reconfigure from the transitory configuration in Figure 8a to the nonorthogonal self‐locking configuration in Figure 8b, in which $\theta_{1}=\theta_{3}=\theta_{4}=0^{\circ },\theta_{2}=-45^{\circ }$. Under this condition, limbs 1 and 3 may still preserve part of the original symmetry, but limbs 2 and 4 no longer maintain the geometric condition required for the twist or translation configurations as described by

(30) $$n_{1}\left(0^{\circ }\right)=-n_{3}\left(0^{\circ }\right),\;n_{2}\left(-45^{\circ }\right)\neq \pm n_{4}\left(0^{\circ }\right).$$

The nonorthogonal self‐locking configuration is therefore governed by geometric constraints. In this representative state, the programming angles satisfy neither the twist‐motion condition nor the coplanar condition for the translation modes. In this case, the upper platform is subjected to a set of resultant constraint moments consisting of one pair of equal and opposite couples together with two additional independent couples, which suppress the admissible global motion of the upper platform. For the orthogonal self‐locking configuration shown in Figure 8c, the programming angles of the actuated joints are $\theta_{1}=\theta_{2}=\theta_{3}=\theta_{4}=45^{\circ }$, and

(31) $$n_{1}\left(45^{\circ }\right)=-n_{3}\left(45^{\circ }\right),\;n_{2}\left(45^{\circ }\right)=-n_{4}\left(45^{\circ }\right).$$

It implies that the constraint couples applied by two nonadjacent limbs are parallel. The joints in those four limbs, therefore, define four distinct planes, respectively. In contrast to the previous nonorthogonal configurations, the corresponding limb planes are not coincident. Instead, the planes associated with limbs 1 and 3 are parallel, and so are those associated with limbs 2 and 4. The adjacent planes become mutually orthogonal. Meanwhile, all limbs simultaneously reach the designed boundary limits of $\theta_{i}=\pm 45^{\circ }$. This orthogonal self‐locking configuration is governed by the combined effect of geometric constraints and mechanical constraints. These boundary constraints suppress further reorientation of the limbs and prevent the upper platform from entering the twist or translation modes.

Apart from the transitory configuration from where the mechanism can switch to different motion modes, home positions, and admissible boundaries are dependent on each motion mode. In the twist motion mode, the mechanism evolves along a helical mode governed by α=2θ. The position with $\theta =0^{\circ }$ and $\alpha =0^{\circ }$ is the home position at which the clockwise and counterclockwise twist branches meet. In each translation mode, the position with $\beta_{i}=0^{\circ }$ is the corresponding home position at which the mechanism implements either lateral or longitudinal planar motion. The physical translation range is reduced from its ideal geometric boundary by finite structural thickness and adjacent‐limb contact.

The above analysis shows that the representative configurations can be identified by a set of programming‐angle relations. These relations determine whether the mechanism admits a finite motion mode or enters a locking mode. The mentioned configuration conditions and motion or locking principles are summarized in Table 2. This summary highlights that the same mechanism can be reprogrammed by changing programming angles $\theta_{1},\theta_{2},\theta_{3},\theta_{4}$. The transitory, twist, and translation configurations correspond to admissible motion states, while the two self‐locking configurations correspond to constrained states. In particular, the orthogonal self‐locking state is distinguished from the twist state by $\alpha =0^{\circ }$, although the four programming angles are equal.

**TABLE 2:** Summary of the configuration conditions and motion or locking principles.

Configuration	Locking or motion condition	Locking or motion principle
Transitory	$\theta_{1}=\theta_{2}=\theta_{3}=\theta_{4}=0^{\circ },\;\alpha =0^{\circ }$	Reference state
Twist	$\theta_{1}=\theta_{2}=\theta_{3}=\theta_{4}=\theta ,\;\theta \neq 0^{\circ },\;\alpha =2\theta$	Helical motion
Translation	$\theta_{1}=-\theta_{2}=\theta_{3}=-\theta_{4}=\pm \theta_{\lim}$	Two 1‐DoF translations
Orthogonal self‐locking	$\theta_{1}=\theta_{2}=\theta_{3}=\theta_{4}=\pm \theta_{\lim},\;\alpha =0^{\circ }$	Geometric and mechanical locking
Nonorthogonal self‐locking	$\exists i:\theta_{i}\neq \theta_{j},\;\forall j\neq i,\;\alpha =0^{\circ }$	Geometric locking

*Note*: $\theta_{i}\in \left[-\theta_{\lim},\theta_{\lim}\right]$, $\theta_{\lim}=45^{\circ }$, $\alpha \in [-90^{\circ },90^{\circ }]$, and i,j∈{1,2,3,4}, i≠j.

## 4 Proof‐of‐Concept Prototype and Actuation

### 4.1 Prototype of the Mechanism

**FIGURE 9:**
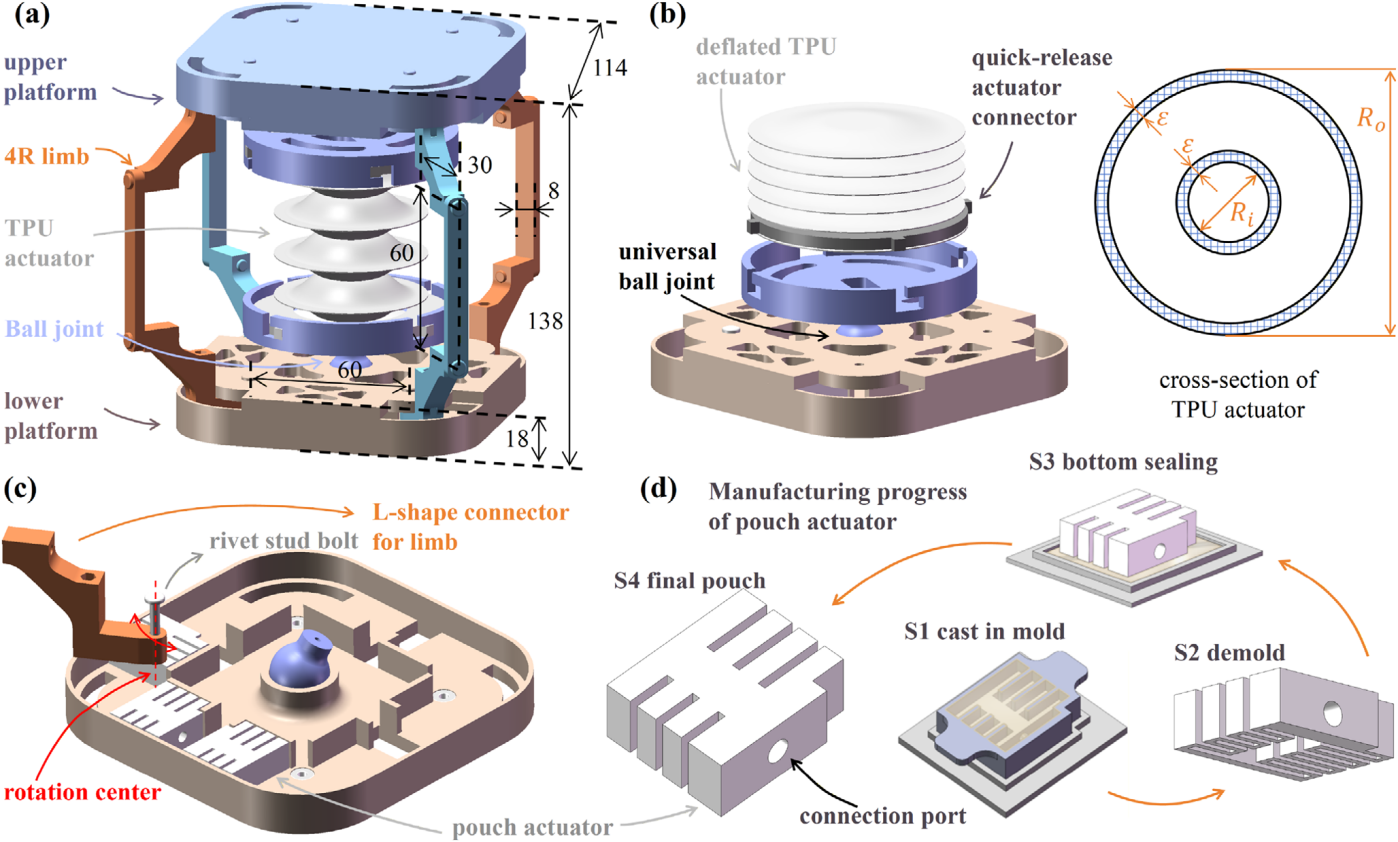
Assembled prototype with actuators. (a) Prototyped mechanism with actuators. (b) Six‐layer TPU actuator with ball‐joint and quick‐release connections. (c) Limb‐programming unit inside the lower platform. (d) Fabrication process of the pouch actuator, including casting, demolding, bottom sealing, and final sealing.

A 3D model of the assembled kirigami‐inspired reconfigurable mechanism is shown in Figure 9a, and all rigid structural components in the proof‐of‐concept prototype were fabricated using a Bambu X1C 3D printer with PLA filament. In the prototype, the lower platform is fixed, while the moving upper platform is connected to the fixed base by four identical serial 4R limbs arranged in a square layout. The four limbs encode the configuration‐dependent constraint pattern through their programmable reorientation, whereas the central multilayer TPU actuator provides the axial input along the global vertical direction. For the prototype, the key geometric parameters introduced in Section 3.1 are specified as l=60mm and d=60mm. The upper and lower platforms were designed as identical square plates with an edge length of 114mm and a thickness of 18mm, yielding an overall prototype height of 138mm. The ideal kinematic model assumes zero‐thickness limbs and therefore predicts the instantaneous twist without considering geometric interference. Under this idealization, the translation mode can, in principle, evolve over a wide range of the limb deflection angle $\beta_{i}$. In the physical prototype, however, the finite width of the limbs introduces collision between adjacent limbs during large‐stroke translation. This finite‐size effect does not alter the instantaneous motion screw derived from the screw‐theoretic model, but it limits the reachable configuration range along that screw. In the present prototype, each limb has a finite width of 8mm, and the maximum admissible limb deflection angle is determined by adjacent‐limb collision. For the current hardware dimensions, this limit is $\beta_{\max}=84.09^{\circ }$.

The multilayer TPU pneumatic actuator is placed between the two platforms, as shown in Figure 9b, to increase the achievable axial force while keeping the module compact. In the current setup, a six‐layer stacked TPU actuator is used. The actuator is connected to the two platforms through a pair of ball joints and rotatable quick‐release connectors. The ball joints are essential because the relative pose between the upper and lower platforms varies across configurations, involving not only axial separation but also configuration‐dependent lateral displacement and platform rotation. Their three rotational degrees of freedom allow the actuator ends to self‐align with these changing boundary conditions, thereby reducing parasitic bending moments, misalignment, and unintended constraint forces at the actuator–platform interfaces. The actuator is fabricated from TPU film with a thickness of 0.3mm and is designed with an inner through‐hole of diameter 20mm for air supply and routing and an outer diameter of 66mm. Adjacent layers are bonded by heat sealing over an annular width ε=6mm at both the inner and outer rims, providing reliable sealing and load transfer across the stack.

Configuration programming is implemented at the base attachments of the limbs as presented in Figure 9c. Each limb is supported by a bearing and mounted to the lower platform via a rivet stud bolt, which defines the local pivot for reorienting the limbs. The reorientation is actuated by a pair of pouch actuators arranged to act on an L‐shaped connector of the limb. When one pouch actuator is pressurized, it expands into the available clearance and elongates in the actuation direction, pushing the L‐shaped connector and generating a controlled rotation about the bolt axis. Using pouch actuation, the limb can be driven in either direction of the programmed angle within the bounded reorientation range. This mechanism enables independent programming of the four limbs, which is the key enabler for switching among twist, bidirectional translation, and self‐locking configurations.

Figure 9d summarizes the fabrication process of the pouch actuators used for limb programming. The actuators are cast from Ecoflex 00‐10 by mixing Part A and Part B at a 1:1 ratio and degassing prior to molding. The mixture is poured into a two‐part mold assembly in which the outer mold is designed higher than the inner core to form a closed top surface after curing (Figure 9d‐1). The demolded intermediate part has an open bottom surface while already containing the internal inflation channel as presented in Figure 9d‐2. The bottom surface is then sealed by placing the part onto a flat plate covered with uncured silicone to form a continuous membrane as shown in Figure 9d‐3. After the final sealing step, an enclosed pouch actuator is obtained (Figure 9d‐4) and used for actuation. A connection port is integrated for pneumatic tubing, and two side cutouts are intentionally introduced to reduce constraints and promote repeatable out‐of‐plane bulging during inflation. The resulting pouch actuator provides a compact and compliant driver for rotating the L‐shaped connector, thereby programming the angles required by the mobility analysis.

### 4.2 Actuation System

The four limbs are equipped with embedded pouch actuators that implement discrete reorientation programs. This architecture realizes a two‐stage open‐loop control strategy. First, the angles are programmed to select a configuration‐dependent constraint set. The central TPU actuator is then actuated to drive the mechanism along the corresponding global motion. Each limb contains two pouch actuators located on the left and right sides of its L‐shape connector. Inflation of a pouch creates a lateral expansion that pushes against the connector and generates a rotation about the rivet stud bolt. Due to the mechanical hard stops in the connector joint, the programmed reorientation saturates at $\pm 45^{\circ }$, providing a repeatable discrete programming variable that matches the configuration programs used in Section 3. Let $u_{iL},u_{iR}\in \left\{0,1\right\}$ in Equation (32) denote the binary activation of the left and right pouch actuators of limb i∈{1,2,3,4}, respectively. The resulting programmed reorientation angle $\theta_{i}$ is modeled as

(32) θi=−45∘,[uiL,uiR]=[1,0]−45∘,[uiL,uiR]=[0,1]−0∘,[uiL,uiR]={[0,0],[1,1]},i∈{1,2,3,4}.

Table 3 summarizes the discrete pouch‐actuation patterns used to program the principal configurations and deployment modes of the mechanism. Here, the binary pair listed for each limb denotes the discrete actuation states of the two pouch actuators associated with that limb. Under the mechanical hard‐stop constraints, [1,0] and [0,1] prescribe $\theta_{i}=-45^{\circ }$ and $\theta_{i}=45^{\circ }$, respectively. And [0,0] and [1,1] correspond to $\theta_{i}=0^{\circ }$, thereby establishing a direct mapping from local actuator states to programmed angles. The transitory configuration corresponds to the default state $\left(\theta_{1}=\theta_{2}=\theta_{3}=\theta_{4}=0^{\circ }\right)$, in which all four limbs remain in the neutral state [0,0]. In addition, the transitory state can also be achieved by simultaneously activating the two pouch actuators on both sides of each limb, $\left[u_{iL},u_{iR}\right]=\left[1,1\right]$ for all i, which drives the limbs toward the middle angle $\theta_{i}=0^{\circ }$ through bilateral actuation. Under axial actuation, this zero‐twist state evolves into the helical twist mode. The two directional translation modes are obtained by alternating the limb‐level actuation states between [1,0] and [0,1], equivalently programming $\theta_{1}=-\theta_{2}=\theta_{3}=-\theta_{4}=\pm 45^{\circ }$, which selects one of the two translation directions. Depending on the selected alternating pattern, the mechanism exhibits either lateral–vertical or longitudinal–vertical translation. Self‐locking can be achieved either globally, by programming all limbs to the same limit state $\left(\theta_{1}=\theta_{2}=\theta_{3}=\theta_{4}=\pm 45^{\circ }\right)$, corresponding to the orthogonal self‐locking configurations I and II, or locally, by reorienting only one limb so that the overall pattern satisfies neither the twist condition nor the translation condition, thereby producing the nonorthogonal self‐locking configuration.

**TABLE 3:** Mapping between discrete pouch‐actuator states and programmed mechanism configurations.

Configuration	Limb 1	Limb 2	Limb 3	Limb 4	Programming angles
Transitory I	[0,0]	[0,0]	[0,0]	[0,0]	$\theta_{1}=\theta_{2}=\theta_{3}=\theta_{4}=0^{\circ }$
Transitory II	[1,1]	[1,1]	[1,1]	[1,1]	$\theta_{1}=\theta_{2}=\theta_{3}=\theta_{4}=0^{\circ }$
Longitudinal‐vertical translation	[1,0]	[0,1]	[1,0]	[0,1]	$\theta_{1}=\theta_{3}=-45^{\circ },\;\theta_{2}=\theta_{4}=45^{\circ }$
Lateral‐vertical translation	[0,1]	[1,0]	[0,1]	[1,0]	$\theta_{1}=\theta_{3}=45^{\circ },\;\theta_{2}=\theta_{4}=-45^{\circ }$
Non‐orthogonal self‐locking	[1,0]	[0,0]	[0,0]	[0,0]	$\theta_{1}=-45^{\circ },\;\theta_{2}=\theta_{3}=\theta_{4}=0^{\circ }$
Orthogonal self‐locking I	[1,0]	[1,0]	[1,0]	[1,0]	$\theta_{1}=\theta_{2}=\theta_{3}=\theta_{4}=-45^{\circ }$
Orthogonal self‐locking II	[0,1]	[0,1]	[0,1]	[0,1]	$\theta_{1}=\theta_{2}=\theta_{3}=\theta_{4}=45^{\circ }$

To switch from one configuration to another, the central TPU actuator first returns the deployed mechanism to the home position of its current motion mode. The pouch actuators are then commanded according to the transitory actuation pattern, which sets $\theta_{1}=\theta_{2}=\theta_{3}=\theta_{4}=0^{\circ }$ and brings the mechanism to the transitory configuration shown in Figure 2a‐1. Subsequently, the target pouch‐actuation pattern is applied to reconfigure the mechanism to the home position of the target motion mode. If the target mode is mobile, the central TPU actuator is then activated to drive the mechanism. For example, switching from the deployed longitudinal–vertical translation configuration as shown in Figure 2b‐3 to the lateral–vertical translation configuration, the central TPU actuator needs to return the mechanism to the home position of the longitudinal–vertical translation mode in Figure 2b‐1 first. The pouch actuators then restore the transitory configuration in Figure 2a‐1 and subsequently apply the target actuation pattern to establish the home position of the lateral–vertical translation mode in Figure 2c‐1. The central TPU actuator can then be activated from this home position to generate the lateral–vertical translation motion as presented in Figure 2 c3.

In the experimental setup, the pneumatic actuation hardware consisted of six 12V DC mini air pumps controlled through a relay‐switching module driven by an Arduino Mega. Two pumps were dedicated to the central multi‐layer TPU actuator, one for axial deployment and another one for recovery. The two pumps were not activated simultaneously, and the Arduino sent digital control signals to the relay module to selectively power the required pump according to the desired actuation stage. The remaining four pumps were used for limb‐level configuration programming through the pouch actuators. The air‐pressure network was distributed to the eight pouch actuators through individually addressable valves, allowing the binary states to be selected independently for each limb. The pouch actuators defined the local constraint pattern of the mechanism, whereas the central TPU actuator provided the global axial input for the corresponding deployment mode. The command signals were entered from a host computer through keyboard input and transmitted to the Arduino Mega, which drove the relay module to switch the pumps on or off. This hardware implementation provides an actuation architecture for realizing the discrete programming states listed in Table 3. Because the programming variable is discrete and terminated by mechanical hard stops, an open‐loop relay‐switched pneumatic architecture is sufficient for the present proof‐of‐concept prototype.

## 5 Experimental validation

This section experimentally validates the its mobility by quantifying the rigid‐body motion of the top platform under different programming angle sets. To reconstruct the platform pose during deployment, an optical motion‐capture system was used to track five retro‐reflective markers attached to the top plate at 120 Hz. The marker set was arranged so that four peripheral markers were placed to form a square, and a fifth marker was placed at the square center. During the experiment, the prototype was placed on a hollow support stand so that the pneumatic tubes could be routed beneath the lower platform without interfering with the motion of the mechanism.

### 5.1 Reconfigurability

**FIGURE 10:**
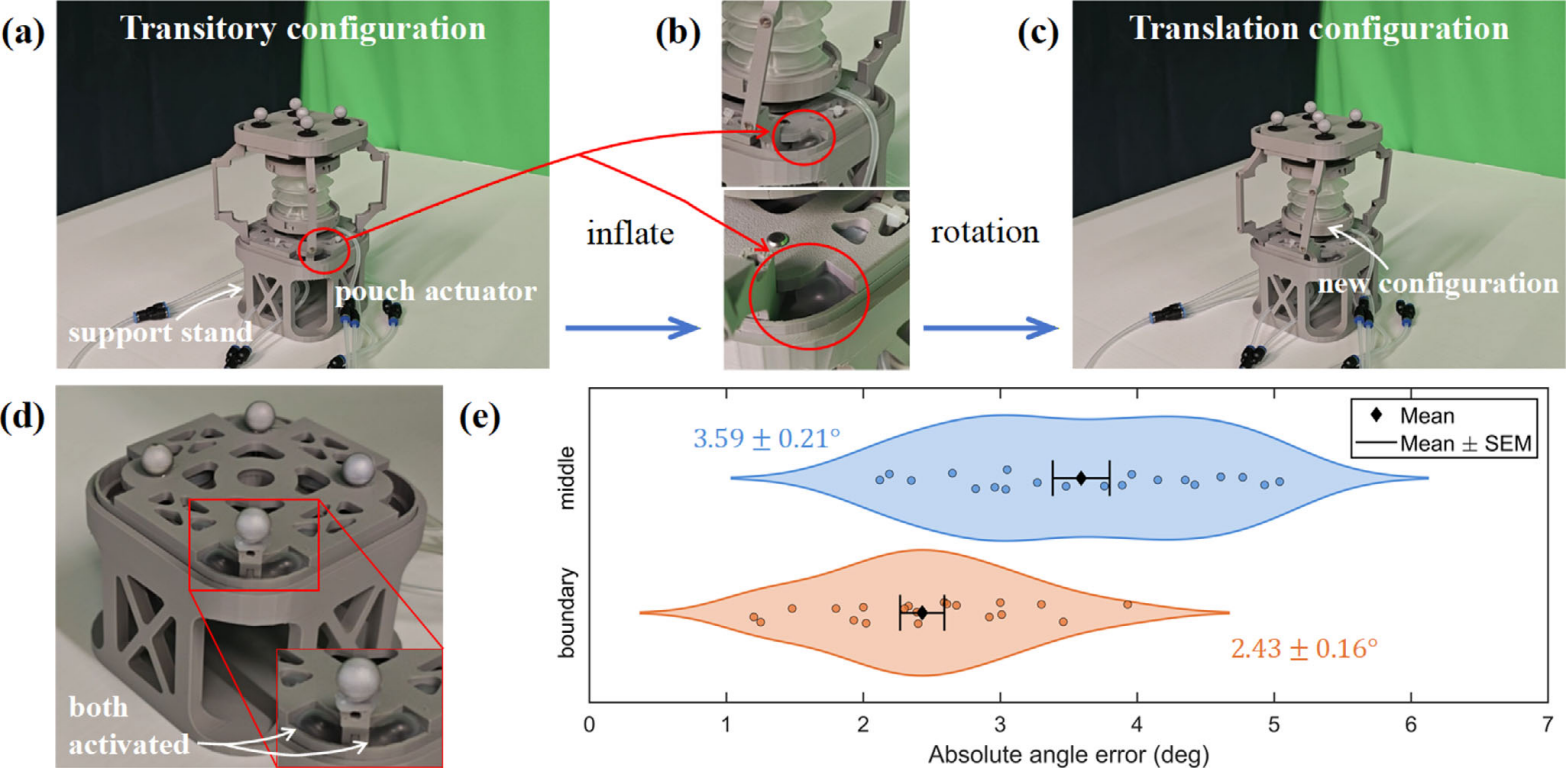
Experimental setup and demonstration of reconfigurability. (a) Transitory configuration and the mechanism mounted on a hollow support stand to provide clearance for pneumatic tube routing. (b) Inflation of the pouch actuators rotates the limb toward the programmed orientation. (c) Programmed configuration after pouch‐actuator actuation. (d) Bilateral pouch‐actuator activation for driving the limb toward the middle target. (e) Repeatability of programming angle setting over 20 repeated trials. The middle target corresponds to $\theta_{i}=0^{\circ }$, and the boundary target corresponds to $|\theta_{i}|=45^{\circ }$. The violin plots show the absolute angle errors relative to the target angles.

A qualitative experiment was first conducted to verify that the prototype can be reliably reprogrammed at the limb level and to visualize the local actuation mechanism responsible for such reconfiguration based on the actuation principle described in Section 4.2. In the transitory configuration, both pouch actuators were inactive, and the limb remained at its neutral orientation, as shown in Figure 10a. Once one pouch actuator was pressurized, the chamber expanded preferentially out of plane and bulged into the available clearance, as highlighted in Figure 10b. This inflation‐induced bulging produced a lateral contact force on the L‐shaped connector and generated a moment about the rivet–stud–bolt axis, thereby rotating the limb toward the selected programmed direction. As the inflation continued, the limb rotation increased progressively until it was bounded by the mechanical stop, leading to a discretely defined orientation, as illustrated in Figure 10c. Pressurizing the pouch actuator on the opposite side resulted in the mirrored reorientation process, enabling bidirectional programming of the same limb.

To further evaluate the repeatability of pouch–actuator‐based limb programming, we conducted repeated angle reprogramming tests using a marker‐based measurement method. Three markers were attached to the fixed platform to define the reference frame, and one marker was attached to the rotating limb to track the programming angle as demonstrated in Figure 10d. Two representative target states were tested. The first was the middle target, where the two pouch actuators on both sides of the limb were activated simultaneously to drive the limb toward $0^{\circ }$, as shown in Figure 10d. The second was the boundary target, where a single‐side pouch actuator was activated to drive the limb toward $\theta_{i}=\pm 45^{\circ }$. Each target state was tested 20 times. The mean absolute angle error for the middle target was defined as $|\theta_{i}|$ and $\left|\right|\theta_{i}|-45^{\circ }|$ for the boundary target. As shown in Figure 10e, black diamond markers indicate the means, and horizontal bars indicate the mean ± Standard Error of the Mean (SEM). The mean absolute angle error for the middle target was $3.59\pm 0.21^{\circ }$, whereas the boundary target showed an absolute angle error of $2.43\pm 0.16^{\circ }$. The middle target showed a larger error than the boundary target because the middle state relies on balanced bilateral pouch actuation, whereas the boundary state is assisted by the designed mechanical limit. The residual angle errors result from pressure imbalance, residual pouch bulging, hinge friction, and marker‐placement uncertainty. These errors perturb the programming angles $\theta_{i}$, which can lead to small deviations in lift‐twist coupling, direction‐dependent offsets in translation, and changes in the constraint‐couple directions in the self‐locking state. Nevertheless, the measured errors remained within a few degrees, confirming that the pouch actuators can repeatedly prepare the limb states required for configuration switching.

This observation confirms that the pouch actuator serves as a local programming element that reorients each limb and modifies the mechanism's internal constraint pattern. After the desired limb‐orientation pattern was established, the central multilayer TPU actuator provided the axial stroke and force required to drive the mechanism into the corresponding global mode, such as twist or directional translation. In this way, the proposed design separates configuration programming from global deployment. The pouch actuators determine the constraint state of the mechanism, whereas the central actuator excites the motion admitted by that state. Therefore, the prototype can switch among different configuration‐dependent modes, which forms the basis for the repeatable functional demonstrations reported below.

### 5.2 Uniaxial Twisting

**FIGURE 11:**
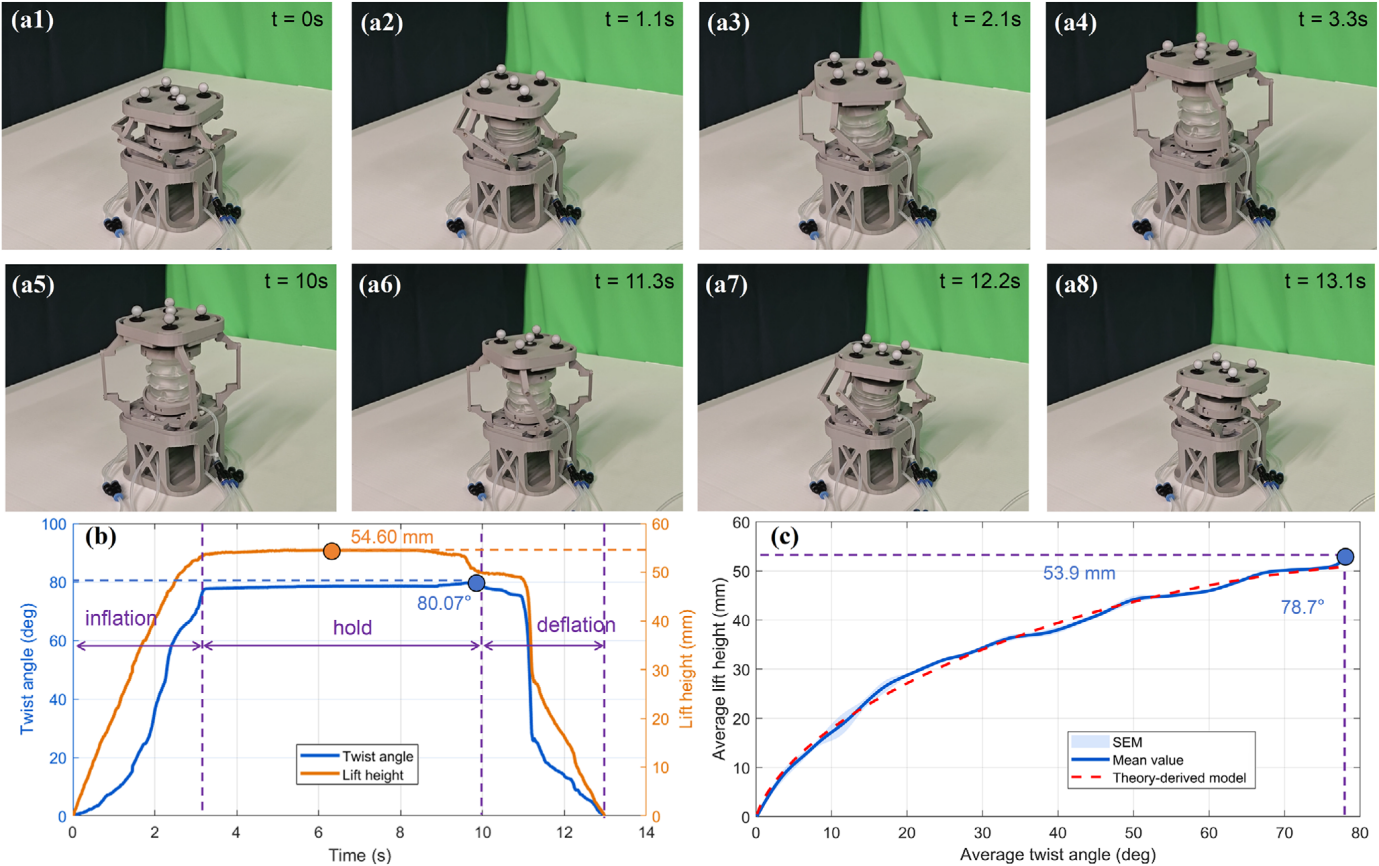
Twist configuration experiment and motion‐capture characterization. (a‐1–a‐8) Representative snapshots during one actuation cycle. (b) Time histories of the measured twist angle α(t) and lift height h(t). (c) Mean lift‐twist coupling reconstructed from 10 monotonic tests, with the SEM band and the theory‐derived fit.

This experiment evaluated whether the prototype exhibits the lift‐twist coupling predicted for the twist mode as analyzed in Section 3.3.2. The motion behavior of this mode is represented by the measured twist angle α(t), lift height h(t), and lift‐twist coupling h(α), as shown in Figure 11. The image sequence in Figure 11a‐1–8 shows one complete actuation cycle, including inflation‐driven deployment, holding near the maximally deployed state, and the recovery process during depressurization. The single‐trial time histories extracted from motion capture are summarized in Figure 11b, which corresponds to the complete actuation cycle illustrated by the snapshots in Figure 11a‐1–8. The twist angle α(t) (blue, left axis) and the lift height h(t) (orange, right axis) are both referenced to the initial frame in Figure 11a‐1. The marker‐based definitions of the two plotted variables are specified to better describe the movement. The two variables α(t) and h(t) were extracted from the motion‐capture data using five markers attached to the upper platform, which are denoted as

(33) $$P_{n}\left(t\right)={\left[x_{n}\left(t\right),\;y_{n}\left(t\right),\;z_{n}\left(t\right)\right]}^{T},\;n\in \left\{0,1,2,3,4\right\}$$

where $P_{0}$ represents the marker located at the center of the upper platform, and $P_{1}$‐$P_{4}$ represent the four peripheral markers. For the twist angle, the horizontal vector from the center marker to each peripheral marker is defined as $r_{k}\left(t\right)={\left[x_{k}\left(t\right)-x_{0}\left(t\right),\;y_{k}\left(t\right)-y_{0}\left(t\right),\;0\right]}^{T}$, with k∈{1,2,3,4}. The twist angle was then calculated by averaging the rotation information from the four peripheral markers

(34) $$\alpha \left(t\right)=\frac{180}{\pi }atan2\left(\frac{1}{4}\sum_{k=1}^{4}e_{z}\cdot \left[r_{k}\left(0\right)\times r_{k}\left(t\right)\right],\frac{1}{4}\sum_{k=1}^{4}r_{k}\left(0\right)\cdot r_{k}\left(t\right)\right).$$

This definition gives the averaged rotation of the upper platform about the base vertical axis, with the four peripheral markers used together to reduce marker‐level measurement variation. The lift height is calculated as the average vertical displacement of the five markers relative to the initial frame:

(35) $$h\left(t\right)=\frac{1}{5}\sum_{n=0}^{4}\left[z_{n}\left(t\right)-z_{n}\left(0\right)\right].$$

With these definitions, the two curves in Figure 11b represent the rotational and vertical responses of the same upper platform throughout one complete actuation cycle. During the first 3.3 s, both quantities increased rapidly as the actuator was inflated. This was followed by a sustained pressurized stage from about t=3.3 to 10.0 s, during which the prototype remained inflated. This indicates that the structure had approached its reachable deformation range under the present actuation condition. In this stage, the lift height remained close to 54 mm and reached its maximum value of $h_{\max}=54.60$ mm at t=6.11 s, whereas the twist angle stayed near $78^{\circ }$ for most of the plateau and reached $\alpha_{\max}=80.07^{\circ }$ at t=9.83 s. The peak values of the two curves did not occur at exactly the same time. After the overall shape had become nearly stable under sustained pressure, the panel connections and frictional contact interfaces within the folded structure can still undergo slow local adjustment. Small local tilting and slight changes in platform posture may therefore introduce subtle variations in the measured lift height and twist angle, which may contribute to the separation of their peak times. After depressurization started at about t=10.0 s, both quantities decreased, and the structure returned toward the twist home position, within the final ∼3 s. The small oscillations superposed on the curves were likely caused by pneumatic pressure fluctuations during switching, local stick–slip, and elastic rebound within the folded structure.

Figure 11c summarizes the lift‐twist coupling over repeated actuation cycles and presents the relation between twist angle α and lift height h. The dataset was constructed from five full inflation‐deflation cycles. For each cycle, one monotonic test was extracted from the inflation stage and another one from the deflation stage, yielding 10 monotonic branches in total. The experimental α‐h relation was represented by the mean curve, with an SEM band, across these 10 tests. The SEM band remained narrow over the measured range, with a maximum of approximately 1.17 mm, indicating that the estimated mean lift–twist relation is stable across the extracted tests. The maximum mean lift height is approximately 53.9 mm near $\alpha =78.4^{\circ }$. To compare the experimental mean relation with the kinematic model, the fitted curve in Figure 11c was generated from the instantaneous screw‐pitch relation derived for the twist mode in Section 3.3.2. Specifically, Equation (19) is used as the theoretical basis for the coupling between axial translation and yaw rotation. The resulting theory‐derived fit closely followed the experimental mean curve over the measured operating range. This agreement indicates that the measured lift–twist coupling is consistent with the twist mode predicted by the screw‐theoretic kinematic model.

### 5.3 The Two 1‐DoF Translations

**FIGURE 12:**
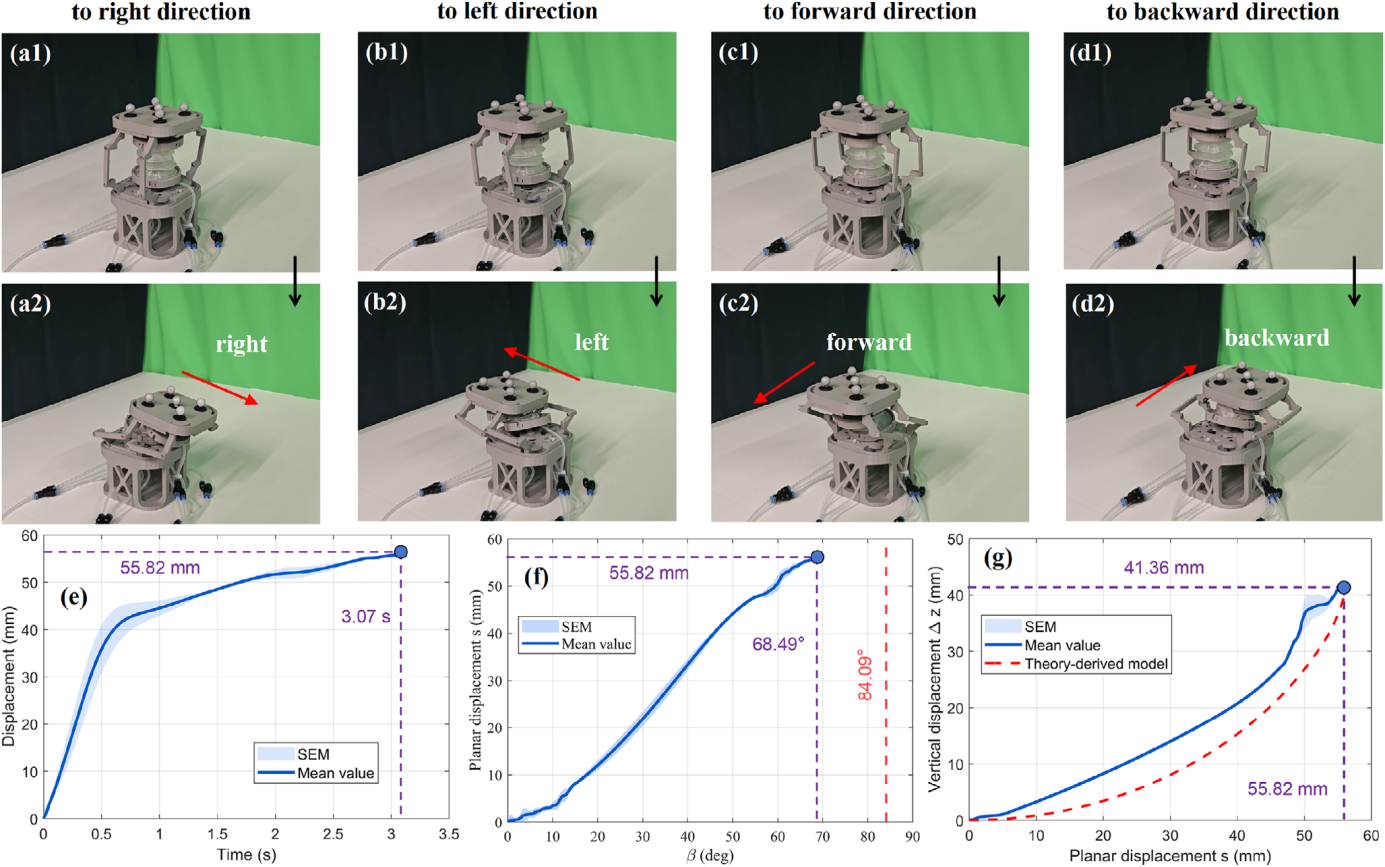
Translation experiment and kinematic characterization. (a‐1,2) Lateral–vertical motion toward the right. (b‐1,2) Lateral‐vertical motion toward the left. (c‐1,2) Longitudinal–vertical motion in the forward direction. (d1,2) Longitudinal–vertical motion in the backward direction. (e) Planar displacement magnitude response over time. (f) Planar displacement s as a function of β, with the limited range bounded by $\beta_{\max}=84.09^{\circ }$. (g) Vertical displacement as a function of planar displacement, compared with the kinematic prediction for the translation modes.

To evaluate the direction‐programmed translation configuration analyzed in Section 3.3.3, four commanded translation motions were tested in the right, left, front, and back directions, as shown in Figure 12a–d. These four commanded motions span two 1‐DoF translation modes–the lateral–vertical and longitudinal–vertical modes–with two opposite directional branches in each mode. The translation modes are represented by the planar displacement of the XY plane s(t), the displacement–deflection relation s(β), and the vertical displacementΔz(s), as shown in Figure 12. In each test, the pouch actuators first change the alternating programming‐angle pattern required for the selected translation configuration, and the central multilayer TPU actuator then supplies the axial input to drive the mechanism along the admitted motion mode. The programmed states shown in Figure 2b‐1,c‐1 are the home positions and geometric bifurcation points of the longitudinal–vertical and lateral–vertical translation configurations, respectively. In the translation branch, the moving platform undergoes pure translation with α and $\theta_{i}$ remaining fixed, and only $\beta_{i}$ changes during the movement. For the physical prototype, the reachable translation range is limited by finite‐size effects. As discussed in Section 4.1, the maximum value for $\beta_{i}$ is $\beta_{\max}=84.09^{\circ }$. The measured marker trajectories were converted into the scalar variables used to compare the four commanded translations in Figure 12e–g. The marker‐averaged centroid of the upper platform is calculated as

(36) $$p_{c}\left(t\right)=\frac{1}{5}\sum_{n=0}^{4}P_{n}\left(t\right)={\left[x_{c}\left(t\right),\;y_{c}\left(t\right),\;z_{c}\left(t\right)\right]}^{T}.$$

The planar displacement s(t) is then defined as the horizontal displacement magnitude of this centroid relative to the initial frame as

(37) $$s\left(t\right)=\sqrt{{\left[x_{c}\left(t\right)-x_{c}\left(0\right)\right]}^{2}+{\left[y_{c}\left(t\right)-y_{c}\left(0\right)\right]}^{2}}.$$

The corresponding vertical displacement is calculated from the same centroid

(38) $$\Delta z\left(t\right)=z_{c}\left(t\right)-z_{c}\left(0\right).$$

With the prototype limb length $l=∥A_{i}B_{i}∥=60$ mm as mentioned in Section 4.1, the angle β used in Figure 12f is inferred from the measured planar displacement as

(39) $$\beta \left(t\right)=\frac{180}{\pi }\sin^{-1}\left(\frac{s(t)}{l}\right).$$

Based on the definition, the averaged curves in Figure 12e–g summarize the shared translation behavior of the two programmed 1‐DoF translation modes. Figure 12e summarizes the planar displacement magnitude response over time. The shaded bands indicate the SEM computed over 16 repeated trials, with four trials performed in each commanded direction. The mean displacement increased rapidly, reaching a maximum value of approximately s=55.82 mm at t=3.07 s. The overall response demonstrated planar repositioning with a mean translation rate of approximately 18.16 mm/s. The SEM showed a maximum of approximately 5.30 mm during the rapid deployment stage at t=0.43 s, where the mean displacement was 31.69 mm. After this transient stage, the SEM decreased and reached 0.061 mm at the final measured point. These results support that the programming‐angle patterns can repeatedly select the intended lateral–vertical and longitudinal–vertical translation configurations.

Figure 12f presents the measured planar displacement as a function of the angle β in Equation (39), and it is used to visualize the progression of the translation state. The maximum β reached in the experiment is $68.49^{\circ }$, corresponding to s=55.82 mm. This value is approximately 81.4% of the hardware‐limited admissible range ($\beta_{max}$), confirming that the measured translation response was obtained within the physically reachable range of the prototype. Figure 12g visualizes the averaged coupling between the vertical displacement and planar translation for the same translation‐configuration dataset and compares to the translation screw relation derived in Section 3.3.3. The mean vertical displacement increased with the planar displacement and reached Δz=41.36 mm at s=55.82 mm. This agreement indicates that the observed planar–vertical coupling is consistent with the translation configuration predicted by the screw‐theoretic kinematic model. The SEM remained relatively small throughout the movement. The largest SEM was approximately 2.71 mm at s=50.50 mm, with a mean vertical displacement of 37.37 mm, while the SEM at the final measured point decreased to 0.389 mm.

Overall, the experiments in Sections 5.2 and 5.3 validate the configuration‐dependent mobility predicted by the screw‐theoretic model. The residual discrepancies between the measured responses and the theoretical predictions mainly result from manufacturing and assembly errors that are not considered in the ideal kinematic formulation. In the theoretical model, the revolute joints are treated as ideal axes, the joint clearance is neglected, and links and panels are assumed to have zero thickness. In the fabricated mechanism, assembly clearance and accumulated tolerances may slightly shift the joint axes and alter the orientations of the limb planes, leading to small pose offsets and direction‐dependent errors. Hinge friction and contact friction may also delay the onset of motion and introduce minor hysteresis during pneumatic actuation. In addition, the finite thickness of the structural components can cause local interference between adjacent limbs, thereby limiting the reachable stroke, especially in translation configurations. These factors do not change the predicted motion modes but reduce the experimentally accessible workspace and account for the residual differences between the measured and theoretical curves.

### 5.4 Load‐Bearing at Self‐Locking States

**FIGURE 13:**
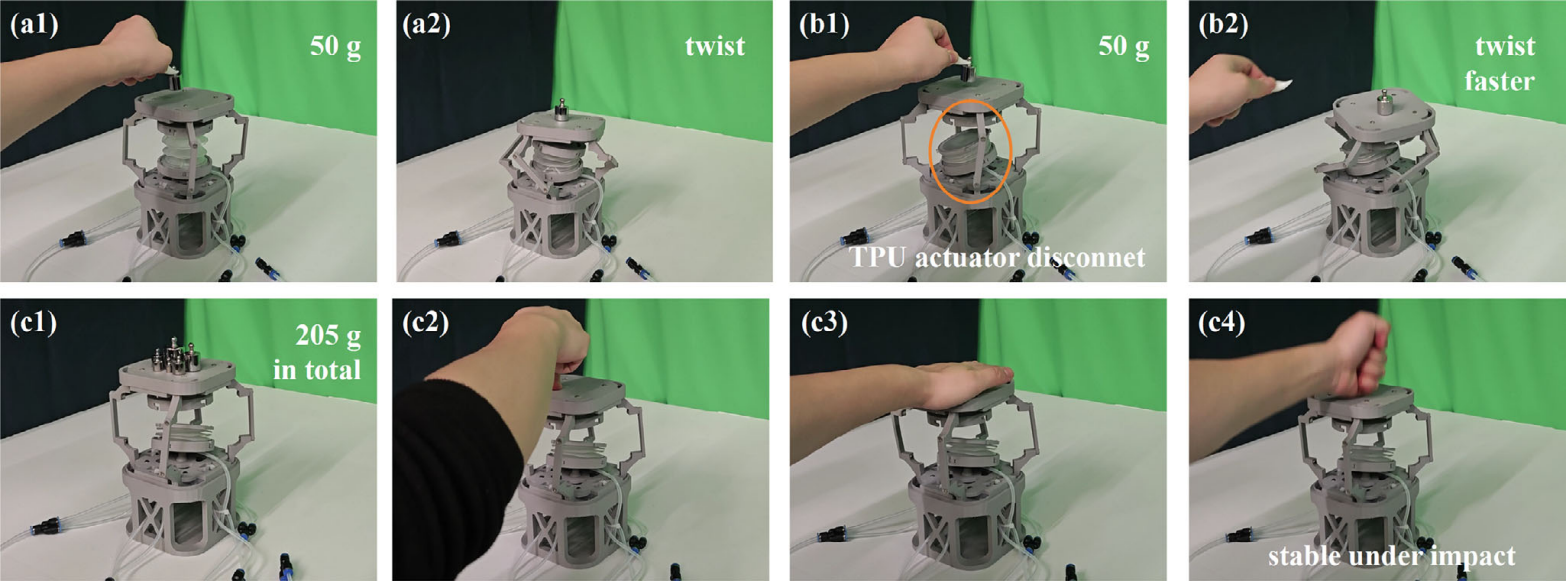
Self‐locking experiment of the prototype. (a‐1,2) In the nonlocked configuration, the structure twists under a small external load (50g). (b1,2) Non‐locked configuration with the TPU actuator disconnected under the same load (50g). (c1) Self‐locking configuration supporting a total payload of 205g. (c2–4) The self‐locking state remains stable under external disturbances without additional energy input.

To evaluate the passive stability of the proposed locking mechanism as analyzed in Section 3.3.4, we conducted self‐locking experiments comparing the twist of the non‐locked configuration with the load‐bearing and disturbance resistance of the self‐locking state. Figure 13a‐1,2 shows the behavior in the nonlocked configuration. With the TPU actuator not activated, a small external load of 50g applied on the upper platform was sufficient to induce a noticeable twisting deformation. This observation indicates that, before locking, the structure remained compliant in torsion and could be perturbed by relatively small off‐axis moments. To further exclude the possibility that the observed twist is dominated by the compliance of the TPU actuator itself, the TPU actuator is disconnected and the same loading condition was repeated (Figure 13b‐1,2). The twisting deformation not only persisted but occurred more rapidly, suggesting that the twist is primarily a configuration‐dependent structural response rather than an actuator‐driven artifact.

After switching the mechanism into the self‐locking configuration, its ability to maintain shape without continuous energy input was tested under increased payload and external disturbances. After incremental loading tests, the prototype sustained a payload of 205g while maintaining a stable geometry, as shown in Figure 13c‐1. Moreover, the self‐locking structure remained stable under external force (Figure 13c‐2–4), including pushing, poking, pressing, and impact‐like disturbances. Notably, these tests were performed without additional actuation input, demonstrating that the self‐locking mechanism can provide a passive, energy‐free holding function and significantly enhance robustness.

**FIGURE 14:**
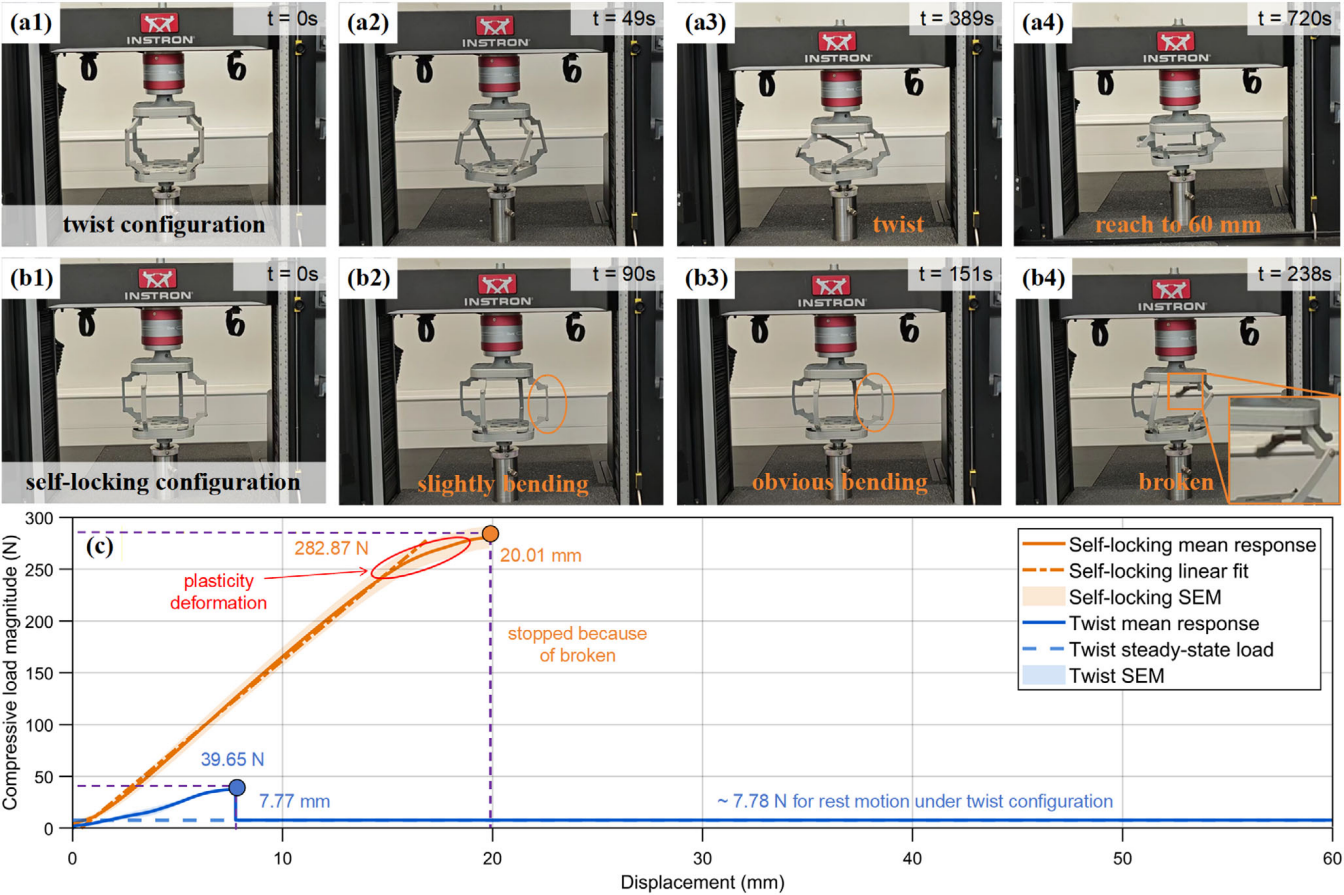
Instron compression test for the twist and self‐locking configurations. (a‐1–4) Representative snapshots showing the twisted state under displacement‐controlled axial compression. (b‐1–4) Representative snapshots showing the self‐locking state under displacement‐controlled axial compression. (c) Force–displacement response of both configurations.

To complement the qualitative demonstrations reported above, displacement‐controlled compression tests were performed on an Instron testing machine (68TM‐10) in both the twist and self‐locking configurations at a loading speed of 5mm per min. Representative camera snapshots are shown in Figure 14a‐1–4,b‐1–4, and the corresponding mean force‐displacement responses are shown in Figure 14c based on four repeated tests, with the shaded regions denoting the SEM. In the twist configuration, the mechanism did not rotate immediately after contact. Instead, the imposed displacement first generated an axial load due to fixture friction and internal hinge friction. As shown in Figure 14c, the mean compressive load rose to a peak of 39.65±3.87N at a displacement of 7.77±1.91mm. After this peak, the structure entered a stable twisting state. The post‐transition twisting load was 7.78±0.99N, as indicated by the steady‐state load line. This response indicates that fixture and hinge friction affect the initial stage of the compression test, followed by a low‐load, repeatable helical deformation stage.

In contrast, the self‐locking configuration exhibited a substantially higher axial load‐bearing capacity. The mean force increased almost linearly with displacement before failure. A linear fit to the mean response gave an apparent stiffness of $16.91\;N\;{\mathrm{mm}}^{-1}$ with $R^{2}=0.998$, as shown in Figure 14c. Across the repeated tests, the self‐locking configuration reached a peak load of 282.87±10.50N at a displacement of 20.01±0.08mm. This peak was taken as the onset of structural failure, which was associated with visible fracture of one limb in the representative test. Compared with the twist configuration, the self‐locking state therefore provided much larger compressive resistance and maintained its integrity until the load approached the structural failure limit of the mechanism members and joints.

To clarify the loading conditions under which the self‐locking claim is valid, we further evaluated the locked configuration under nonaxial loading. The axial compression test described above characterizes the resistance of the locked state along the central axis passing through the geometric centers of the upper and lower platforms. Since the locked configuration constrains the relative axial displacement between the two platforms, axial compression and axial tension act along the same constrained degree of freedom. Therefore, the self‐locking state also suppresses axial tensile displacement within the structural strength limit of the prototype.

**FIGURE 15:**
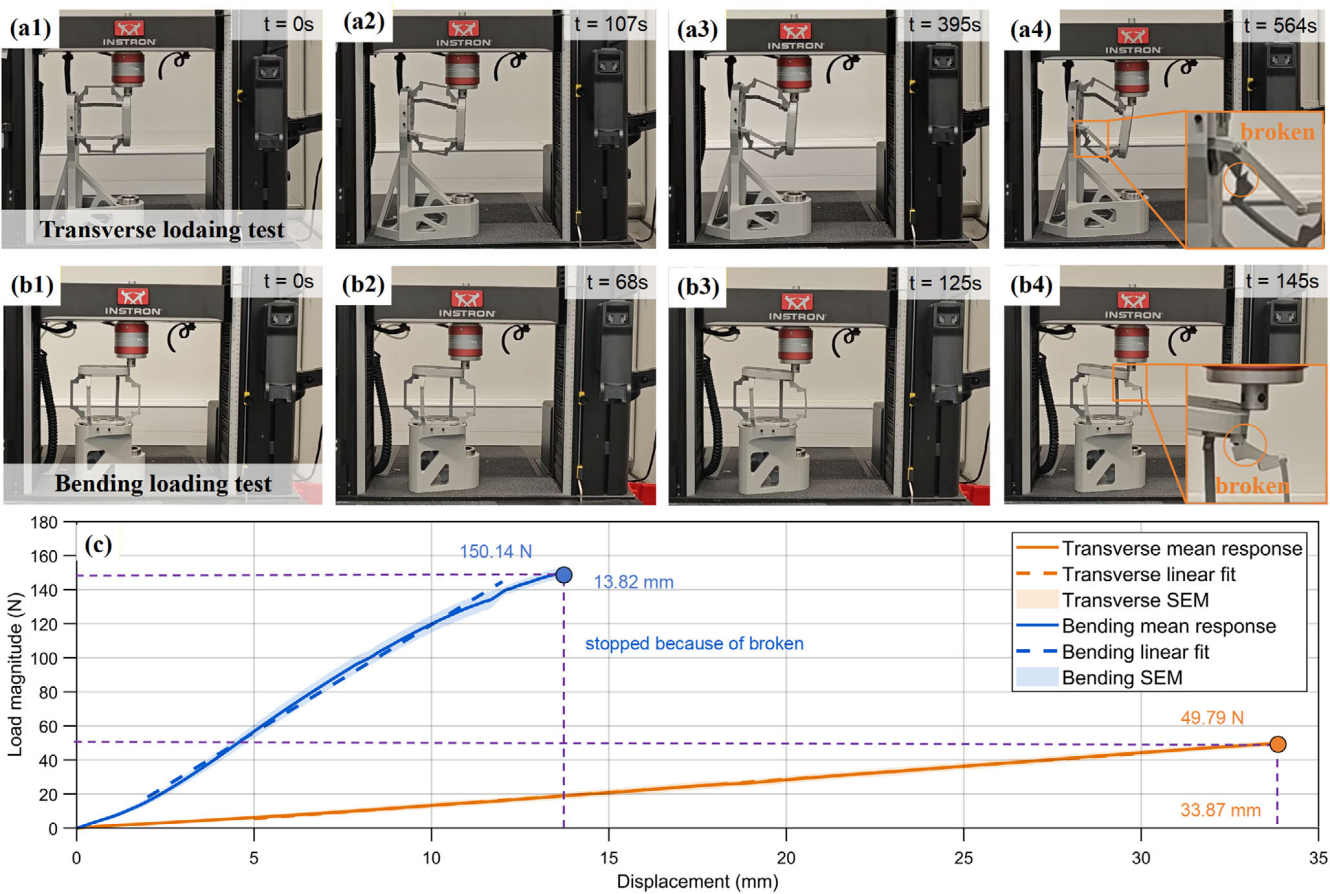
Transverse and bending loading tests of the self‐locking configuration. (a‐1–4) Representative snapshots of the transverse loading test. (b‐1–4) Representative snapshots of the bending loading test, in which the transverse force was applied eccentrically to induce a bending moment. (c) Mean force–displacement responses with SEM bands for the transverse and bending loading tests from four repeated tests. The linear fits are shown to illustrate the approximate load‐displacement trends prior to structural failure.

Two additional displacement‐controlled tests were conducted to examine nonaxial load resistance, and each loading condition was tested four times. In the transverse loading test, the mechanism axis was rotated by $90^{\circ }$ and a transverse force was applied to the upper platform as shown in Figure 15a‐1–4). This loading condition evaluates the lateral load‐bearing capability of the locked state. In the bending loading test as presented in Figure 15b‐1–4, the transverse force was applied at the midpoint of one edge of the upper platform, thereby inducing a bending moment together with transverse shear. As illustrated in Figure 15c, the locked configuration resisted both transverse loading and bending loading until structural failure of the prototype. In the transverse loading test, the mean force reached 49.79 ± 1.54 N at a displacement of 33.87 mm. A linear fit over the loading range from 5 to 33 mm gave a transverse load–displacement stiffness of 1.54 N ${\mathrm{mm}}^{-1}$ with $R^{2}=0.9997$. In the bending loading test, the plotted mean response reached 150.14 ± 3.81 N at a displacement of 13.82 mm. A linear fit over the range from 2 to 12 mm gave a load–displacement stiffness of 12.66 N ${\mathrm{mm}}^{-1}$ with $R^{2}=0.994$ for bending loading.

The self‐locking configuration provided passive resistance to axial compression, bending, and transverse loading until the failure limit of the fabricated prototype was reached. Among the three tested conditions, axial compression exhibited the highest peak force and apparent stiffness, reaching 282.87±10.50N and $16.91\;N\;{\mathrm{mm}}^{-1}$, respectively. This peak force was approximately 1.88 times the force measured under bending loading and 5.68 times the force measured under transverse loading. Bending loading produced a peak force of 150.14±3.81N with an apparent stiffness of $12.66\;N\;{\mathrm{mm}}^{-1}$, whereas transverse loading reached 49.79±1.54N with a substantially lower stiffness of $1.54\;N\;{\mathrm{mm}}^{-1}$. The higher resistance under axial compression can be attributed to the load‐transfer path established by the geometry of the orthogonal self‐locking configuration. A centered axial load preserves the fourfold symmetry of the mechanism and is distributed approximately evenly through the four limbs, producing a predominantly direct compressive load path with limited parasitic bending. In comparison, bending loading introduces a combined moment and shear, causing asymmetric load distribution and higher local stresses in individual limbs. Transverse loading is resisted primarily through limb flexure, joint compliance, and contact constraints, resulting in greater deformation and lower apparent stiffness. These different load‐transfer mechanisms are consistent with the experimentally observed order of axial, bending, and transverse load resistance. Torsion was not treated as a resisted load case because a moment about the central axis directly activates the twisting and configuration‐switching pathway and may therefore unlock or reconfigure the mechanism. The tested prototype uses PLA‐printed rigid parts. Therefore, the measured load resistance in the self‐locking state is limited by the fabricated structural parts and joints. Fabricating the same geometry using materials with higher stiffness or reinforced printed materials could further increase the load‐bearing capacity without changing the constraint‐programming principle.

## 6 Discussion and Conclusions

This paper presents a programmable kirigami‐inspired mechanism that integrates multiple motion modes and self‐locking within a single rigid‐foldable mechanism. By reorienting programming joints and subsequently changing geometric constraints exerted on the platform by the four identical kinematic limb chains, the mechanism reconfigures its internal constraint set to switch among transitory, twist, direction‐programmed translation, and energy‐free self‐locking configurations without mechanical reconstruction. A unified screw‐theoretic framework establishes the relationship between configuration programming and the resulting motion screw system, providing a systematic understanding of the underlying mobility transitions. This reconfigurable folding capability makes the mechanism suitable as a compact functional unit for adaptive robotic systems, deployable structures, morphing supports, and biomedical devices, where controlled motion switching, compact deployment, and passive holding are required.

To benchmark the proposed metamorphic mechanism against existing related work, Table 4 provides a qualitative comparison with representative systems in terms of functional capabilities. In this comparison, translation is defined as a plane‐preserving relative displacement in which the upper and lower reference planes remain parallel, while their relative position changes without relative rotation. Accordingly, bending motions are not classified as translations. Under these definitions, the proposed mechanism is distinguished not only by integrating twist, plane‐preserving translation, and self‐locking configurations within a single structure, but also by combining these functions with reconfigurability and rigid‐foldability. Although several previous systems demonstrate reconfigurability, multimodal deformation, or passive stabilization, these capabilities are often achieved through soft‐body deformation, additional actuation modules, multistable material responses, or task‐specific structural additions. In contrast, the proposed mechanism realizes distinct functional modes by reorienting identical limb chains. This constraint‐reconfiguration strategy establishes a direct correspondence between configuration programming and the resulting motion screw system, thereby providing a compact and analytically tractable route to multifunctional deployable mechanisms.

**TABLE 4:** Comparison with representative works in functional capabilities.

Systems	Reconfigurable	Rigid‐foldable	Twist	Translation	Self‐locking
This work	Y	Y	Y	Y	Y
Origami structure with Kresling pattern [22]	N	Y	Y	N	N
Twisting modules [27, 28]	N	N	Y	N	N
Kresling‐based deformation module [30]	Y	N	Y	N	N
Kresling‐derived multistable continuum units [26]	Y	N	Y	N	Y
Programmable inflatable Kresling actuator [33]	Y	N	Y	N	N
Modular chiral origami [29]	Y	N	Y	N	N
Modular origami‐inspired pneumatic blocks [34]	Y	N	N	N	N
Programmable origami‐inspired modules [35]	Y	N	Y	N	N
Self‐locking origami [42]	N	Y	N	Y	Y
Origami exoskeletons [45]	Y	Y	N	Y	Y
Bennett Plano‐Spherical hybrid linkage [6]	Y	Y	Y	Y	N
Reconfigurable parallel mechanism [7]	Y	Y	Y	Y	N
Polyhedral multi‐facet soft robot [8]	Y	N	Y	Y	N

*Note*: Y and N indicate that the corresponding capability is present or absent, respectively, in the reported system.

The current proof‐of‐concept prototype also has several practical limitations that require further investigation. In the translation configuration, finite thickness and clearance cause contact between adjacent limbs before the ideal geometric extreme is reached, thereby limiting the achievable sliding stroke. In practice, residual bulging after depressurization (e.g., trapped air and viscoelastic recovery) can persist, preventing the limb from reaching the theoretical programming limits and reducing the effective motion range. The repeated limb programming tests also indicate that pouch actuator based reprogramming introduces a small but measurable angular error. Such programming errors can propagate to the programmed motion by perturbing the programmed angles, leading to small deviations in the motions. In addition, because the mechanism contains multiple revolute joints, its performance can be affected by joint clearance, tolerance accumulation, and axis misalignment. These errors may shift the joint axes and perturb the orientations of the limb planes, leading to small pose offsets, direction‐dependent motion errors, and reduced motion accuracy. They may also affect configuration switching and self‐locking because shifted joint axes alter the constraint relations that govern the transition between mobile and locked states. Future prototypes could reduce these errors through improved pressure regulation, more symmetric pouch fabrication, reduced hinge friction, and closed‐loop sensing of programming angles.

Building on the present proof‐of‐concept study, several future research directions could further enhance the capabilities and practical applicability of the proposed mechanism. A direction is the integration of compliant hinges to reduce thickness‐induced interference while preserving the intended rigid‐body kinematics. A second direction is autonomous configuration switching through closed‐loop control. The present prototype employs discrete, open‐loop pneumatic programming, with the programming angles bounded by mechanical stops. Future systems could integrate feedback of the limb programming angles, platform pose, actuator pressure, and contact states to improve the accuracy and robustness of configuration switching. Coupling this feedback with configuration‐transition planning could enable reliable switching among programmed motion modes under external loads and environmental disturbances.

Beyond improving the performance of an individual mechanism, the proposed concept also lends itself naturally to modular robotic systems. Owing to its well‐defined rigid interfaces and programmable motion modes, the proposed unit provides a versatile building block for larger reconfigurable structures. Multiple units could be arranged in serial, planar, or lattice architectures to achieve extended workspace, distributed shape adaptation, and task‐specific reconfiguration. Realizing such multi‐unit systems will require further investigation of mechanical integration, load transfer, and coordinated configuration control. Collectively, these offer a pathway toward scalable reconfigurable robotic and deployable systems that build upon the proposed constraint‐programming principle.[Supplementary-material advs76798-supl-0001]

## Supporting information


**Supporting File**: advs76798‐sup‐0001‐VideoS1‐S2.zip.

## Data Availability

Data sharing not applicable to this article as no datasets were generated or analyzed during the current study.

## References

[1] Z. Chen , Q. Chen , G. Jia , and J. S. Dai , “Sylvester's Dialytic Elimination in Analysis of a Metamorphic Mechanism Derived from Ladybird Wings,” Mechanism and Machine Theory 179 (2023): 105102.

[2] C. Wang , H. Guo , R. Liu , Z. Deng , Y. Chen , and Z. You , “Reconfigurable Origami‐Inspired Multistable Metamorphous Structures,” Science Advances 10, no. 22 (2024): eadk8662. 38809983 10.1126/sciadv.adk8662 PMC11135397

[3] X. Chai , X. Kang , D. Gan , H. Yu , and J. S. Dai , “Six Novel 6R Metamorphic Mechanisms Induced from Three‐Series‐Connected Bennett Linkages that Vary among Classical Linkages,” Mechanism and Machine Theory 156 (2021): 104133.

[4] L. Nurahmi and D. Gan , “Reconfiguration of a 3‐(RR) PS Metamorphic Parallel Mechanism Based on Complete Workspace and Operation Mode Analysis,” Journal of Mechanisms and Robotics 12, no. 1 (2020): 011002.

[5] A. K. Brooks , S. Chakravarty , M. Ali , and V. K. Yadavalli , “Kirigami‐Inspired Biodesign for Applications in Healthcare,” Advanced Materials 34, no. 18 (2022): 2109550. 10.1002/adma.202109550 35073433

[6] K. Zhang and J. S. Dai , “Screw‐System‐Variation Enabled Reconfiguration of the Bennett Plano‐Spherical Hybrid Linkage and Its Evolved Parallel Mechanism,” Journal of Mechanical Design 137, no. 6 (2015): 062303.

[7] J. Wei , C. Qiu , and J. S. Dai , “Configuration Switch and Path Selection between Schönflies Motion and Non‐Schönflies Motion Based on Quotient Manifold of Novel Reconfigurable Parallel Mechanisms,” Mechanism and Machine Theory 180 (2023): 105153.

[8] Y. Wu , N. Huang , S. Tang , et al., “A Multi‐Facet‐Effector Soft Robot in Polyhedral Configuration for Multidirectional Function Reuse,” Mechanism and Machine Theory 217 (2025): 106240.

[9] Y. Zhang , C. Gao , P. Xu , and B. Li , “Type Synthesis of Deployable and Symmetrical Single‐Loop Mechanisms for Constructing Aerospace Platforms,” Mechanism and Machine Theory 181 (2023): 105212.

[10] D. Rus and M. T. Tolley , “Design, Fabrication and Control of Origami Robots,” Nature Reviews Materials 3, no. 6 (2018): 101–112.

[11] L.‐W. Zhao , Y. F. Wu , Y. Fan , and Y. Guo , “Kirigami‐Inspired Linearly Polarized Reconfigurable 3‐D Frequency‐Selective Surface with Controllable Resonant Behavior,” IEEE Transactions on Microwave Theory and Techniques 72, no. 9 (2024): 5193–5203.

[12] Y. Zheng , K. Chen , W. Yang , et al., “Kirigami Reconfigurable Gradient Metasurface,” Advanced Functional Materials 32, no. 5 (2022): 2107699.

[13] R. Baines , S. K. Patiballa , J. Booth , et al., “Multi‐Environment Robotic Transitions Through Adaptive Morphogenesis,” Nature 610, no. 7931 (2022): 283–289. 36224418 10.1038/s41586-022-05188-w

[14] J. Sun , E. Lerner , B. Tighe , C. Middlemist , and J. Zhao , “Embedded Shape Morphing for Morphologically Adaptive Robots,” Nature Communications 14, no. 1 (2023): 6023. 10.1038/s41467-023-41708-6 PMC10533550 37758737

[15] K. Zhang , Y. Zhu , C. Lou , P. Zheng , and M. Kovač , “A Design and Fabrication Approach for Pneumatic Soft Robotic Arms Using 3D Printed Origami Skeletons,” in 2019 2nd IEEE International Conference on Soft Robotics (RoboSoft) (2019), 821–827.

[16] Z. Zhang , G. Chen , H. Wu , L. Kong , and H. Wang , “A Pneumatic/Cable‐Driven Hybrid Linear Actuator With Combined Structure of Origami Chambers and Deployable Mechanism,” IEEE Robotics and Automation Letters 5, no. 2 (2020): 3564–3571.

[17] W. Ding , Z. Zhang , Z. Zhao , et al., “Multiband Switchable Microwave Absorbing Metamaterials Based on Reconfigurable Kirigami–Origami,” Advanced Functional Materials 36 (2025): e15206.

[18] Q. Ze , S. Wu , J. Nishikawa , et al., “Soft Robotic Origami Crawler,” Science Advances 8, no. 13 (2022): eabm7834. 35353556 10.1126/sciadv.abm7834 PMC8967224

[19] P. Sareh , P. Chermprayong , M. Emmanuelli , H. Nadeem , and M. Kovac , “Rotorigami: A Rotary Origami Protective System for Robotic Rotorcraft,” Science Robotics 3, no. 22 (2018): eaah5228. 33141756 10.1126/scirobotics.aah5228

[20] L. Lu , S. Leanza , and R. R. Zhao , “Origami With Rotational Symmetry: A Review on Their Mechanics and Design,” Applied Mechanics Reviews 75, no. 5 (2023): 050801.

[21] K. Zhang , C. Qiu , and J. S. Dai , “Helical Kirigami‐Enabled Centimeter‐Scale Worm Robot With Shape‐Memory‐Alloy Linear Actuators,” Journal of Mechanisms and Robotics 7, no. 2 (2015): 021014.

[22] C. Jianguo , D. Xiaowei , Z. Ya , F. Jian , and T. Yongming , “Bistable Behavior of the Cylindrical Origami Structure With Kresling Pattern,” Journal of Mechanical Design 137, no. 6 (2015): 061406.

[23] T. Liu , Y. Wang , and K. Lee , “Three‐Dimensional Printable Origami Twisted Tower: Design, Fabrication, and Robot Embodiment,” IEEE Robotics and Automation Letters 3, no. 1 (2017): 116–123.

[24] D. Jeong and K. Lee , “Design and Analysis of an Origami‐Based Three‐Finger Manipulator,” Robotica 36, no. 2 (2018): 261–274.

[25] W. Fan , J. Wang , H. Zhang , et al., “SPARC: A Soft, Proprioceptive, Agile Robot for 3D Climbing and Exploration with Precise Trajectory Following,” Advanced Science 12, no. 41 (2025): e10382. 40946185 10.1002/advs.202510382 PMC12591128

[26] X. Wang , Y. Wang , H. Qu , et al., “Coupled Leaf‐Like and Kresling Patterns for Origami‐Inspired Soft Continuum Robots,” International Journal of Mechanical Sciences 303 (2025): 110620.

[27] Z. Jiang and K. Zhang , “A Novel Torsional Actuator Augmenting Twisting Skeleton and Artificial Muscle for Robots in Extreme Environments,” in 2021 IEEE International Conference on Robotics and Automation (ICRA, 2021), 9318–9324.

[28] Z. Jiang and K. Zhang , “Modeling, Optimization, and Control of a Variable Stiffness Pneumatic Rotary Joint With Soft‐Rigid Hybrid Twisting Modules,” Mechanism and Machine Theory 205 (2025): 105899.

[29] T. Zhao , X. Dang , K. Manos , et al., “Modular Chiral Origami Metamaterials,” Nature 640, no. 8060 (2025): 931–940. 40269282 10.1038/s41586-025-08851-0

[30] C. Zhang , Z. Zhang , Y. Peng , et al., “Plug & Play Origami Modules with All‐Purpose Deformation Modes,” Nature Communications 14, no. 1 (2023): 4329. 10.1038/s41467-023-39980-7 PMC10356792 37468465

[31] C. H. Belke and J. Paik , “MORI: A Modular Origami Robot,” IEEE/ASME Transactions on Mechatronics 22, no. 5 (2017): 2153–2164.

[32] J. Huang , J. Zhou , Z. Wang , et al., “Modular Origami Soft Robot With the Perception of Interaction Force and Body Configuration,” Advanced Intelligent Systems 4, no. 9 (2022): 2200081.

[33] S. P. M. Babu , R. Das , B. Mazzolai , and A. Rafsanjani , “Programmable Inflatable Origami,” in 2023 IEEE International Conference on Soft Robotics (RoboSoft) (IEEE, 2023), 1–6.

[34] Y. Park , J. Kang , and Y. Na , “Reconfigurable Shape Morphing With Origami‐Inspired Pneumatic Blocks,” IEEE Robotics and Automation Letters 7, no. 4 (2022): 9453–9460.

[35] Z. Chen , B. Tighe , and J. Zhao , “Origami‐Inspired Modules Enable a Reconfigurable Robot With Programmable Shapes and Motions,” IEEE/ASME Transactions on Mechatronics 27, no. 4 (2022): 2016–2025.

[36] S. Gao , J. Zhang , R. Zhang , et al., “Tri‐Prism Origami Enabled Soft Modular Actuator for Reconfigurable Robots,” Soft Robotics 12, no. 4 (2025): 477–487. 39815957 10.1089/soro.2024.0112

[37] J. Yi , X. Chen , C. Song , et al., “Customizable Three‐Dimensional‐Printed Origami Soft Robotic Joint With Effective Behavior Shaping for Safe Interactions,” IEEE Transactions on Robotics 35, no. 1 (2018): 114–123.

[38] Y. Sun , D. Li , M. Wu , et al., “Origami‐Inspired Folding Assembly of Dielectric Elastomers for Programmable Soft Robots,” Microsystems & Nanoengineering 8, no. 1 (2022): 37. 35450326 10.1038/s41378-022-00363-5 PMC8971403

[39] J. Wu , M. Wu , C. Wang , and G. Xie , “Monolithic Programmable Fabric‐Stacking Enables Multifunctional Soft Robots,” IEEE Transactions on Robotics 41 (2025): 4810–4828.

[40] R. Montazeri , H. D. S. Oliveira , X. Li , et al., “Monolithic 3D Printing of Origami‐Inspired Soft Robotics from Sustainable Bio‐Based Resin,” Advanced Science 13 (2026): e20529. 41566801 10.1002/advs.202520529 PMC13042658

[41] H. Fang , S.‐C. A. Chu , Y. Xia , and K.‐W. Wang , “Programmable Self‐Locking Origami Mechanical Metamaterials,” Advanced Materials 30, no. 15 (2018): 1706311. 10.1002/adma.201706311 29513374

[42] S.‐J. Kim , D.‐Y. Lee , G.‐P. Jung , and K.‐J. Cho , “An Origami‐Inspired, Self‐Locking Robotic Arm That can be Folded Flat,” Science Robotics 3, no. 16 (2018): eaar2915. 33141746 10.1126/scirobotics.aar2915

[43] J.‐K. Kim , S. H. Ahn , S.‐P. Jung , et al., “Double‐Layer Self‐Locking Origami Based on Opposite Folding Motion,” Soft Robotics 13 (2025): 21695172251401337, 10.1177/21695172251401337. 41335104

[44] F. Liu , T. Terakawa , J. Lin , and M. Komori , “Design of Modular Deployable Structure With Programmable Multistability,” International Journal of Mechanical Sciences 288 (2025): 110037.

[45] K. Fu , X. Wu , S. Yu , et al., “Origami Exoskeletons for Enhanced Soft Robotic Manipulation,” Science Advances 11, no. 31 (2025): eadv6629. 40737404 10.1126/sciadv.adv6629 PMC12309676

